# Eco-innovative dyeing of cotton with upcycled pineapple peel waste-derived natural dye

**DOI:** 10.1098/rsos.251200

**Published:** 2025-11-26

**Authors:** Kasindu Pramod, Gayara Perera, Nethmini Wijesundara, Nuwan De Silva

**Affiliations:** ^1^ Sri Lanka Institute of Nanotechnology, Nanotechnology & Science Park Mahenwatta, Pitipana, Homagama 10200, Sri Lanka; ^2^ Department of Physical Sciences & Technology, Faculty of Applied Sciences, Sabaragamuwa University of Sri Lanka, PO Box 02, Belihuloya, Sri Lanka

**Keywords:** natural dye, cotton, eco-textile, pineapple peel, fastness properties

## Abstract

Pineapple (*Ananas comosus*) peel, an abundant agro-industrial by-product, offers significant potential as a sustainable natural dye for textile applications. This study optimized colourant extraction using an alkaline–water system and evaluated dyeing performance on cotton fabrics. Statistical optimization through four-factor linear regression identified optimal conditions: mass-to-liquid ratio of 1 : 10, 12% NaOH, 80°C and 2 h extraction time, yielding 35–40% colourant, significantly outperforming organic solvents (<1%). High-performance liquid chromatography analysis revealed distinctive polyphenolic profiles between alkaline and water extraction methods, demonstrating altered compound distribution and enhanced extraction efficiency under alkaline conditions. Thermogravimetric analysis revealed dye stability up to 160°C, while particle size analysis showed a mean size of 266 nm, enabling effective fibre penetration. Quantitative dyeing evaluation demonstrated 43.05% exhaustion, 27.76% total fixation efficiency and 0.64 fixation ratio. Colorimetric analysis revealed significant mordant-dependent variations, with tannic acid achieving superior colour strength (*K*/*S* = 21.4) compared to metallic mordants: zinc sulfate, alum and ammonium ferrous sulfate. CIELab coordinates confirmed successful dye uptake (L* = −6.92 to −12.83). Post-mordanting with zinc sulfate achieved excellent fastness properties: wash (4–5), light (4) and rubbing (4) fastness. Cationization minimized electrostatic repulsion between cotton and anionic dye molecules, enhancing dye absorption. The findings demonstrate pineapple peel waste viability as a cost-effective natural dye with quantified performance metrics supporting commercial feasibility and circular economy principles in sustainable textile manufacturing.

## 1. Introduction

In the recent past, there has been a rising global concern about the negative environmental externalities imposed by the textile industry. The heavy reliance of the textile industry on synthetic colourants derived from petrochemicals while offering a broader colour spectrum, superior fastness [1] and cost advantages has raised significant environmental concerns due to their hazardous production processes and non-biodegradability [2,3]. The production of many synthetic dyes involves aromatic amines, heavy metals and salts, which are often difficult to degrade and require advanced treatment systems to remove from effluents. These chemicals not only pose significant health risks to labour and neighbourhoods but also result in wastewater streams with high chemical oxygen demand, heavy metal content, and persistent organic pollutants. Such wastewater is difficult to treat effectively and contributes to long-term ecological damage [4].

Natural dyes, once widely used in pre-industrial dyeing traditions, experienced a decline in usage with the dawn of synthetic dyes in the early twentieth century [5]. Despite their historical decline, natural dyes are now receiving renewed interest due to their biodegradable nature, lower toxicity and potential functional benefits. Critically, many natural dyes also originate from renewable resources, especially agro-waste, enabling a circular approach to colourant sourcing. Sustainable green technologies are gaining increasing popularity in the textile industry due to their potential to reduce the environmental and health hazards posed by synthetic dyes and finishing chemicals [4]. Recent studies have emphasized the importance of reintroducing natural dyes by addressing their limitations through mordanting, fibre pre-treatments and optimized extraction protocols [5].

Various plant parts such as leaves [6], roots [7], flowers [8], bark [9], seeds [10], seed kernels [11], peels [12] and fruits [13] are utilized in natural dyeing, offering a diverse palette of eco-friendly colourants [4]. As researchers explore alternatives, the focus has shifted to replacing synthetic chemicals with green resources without compromising production quality or efficiency [14]. Beyond their primary function of colouration, natural dyes have demonstrated additional functional properties, such as flame retardation [15], antibacterial potency [16], UV protection [17] and deodorizing capabilities [4]. Despite these benefits, their limited availability, high processing costs, low dye adsorption, shade reproducibility and poor fastness properties hinder their widespread application in textiles. Still, it is essential to note that natural dyeing often requires careful optimization to overcome drawbacks compared to synthetic dyes. These limitations are not universal but depend heavily on dye structure, extraction technique, fiber–dye compatibility and mordanting systems. Therefore, to establish natural dyes as commercially viable alternatives, it is essential to demonstrate not only their safety and sustainability but also competitive performance in terms of dye uptake, shade reproducibility and functional properties on industrially relevant fibres like cotton [14].

Agriculture, a cornerstone of the global economy, generates vast quantities of by-products and waste [4]. These by-products account for about 30% of global agricultural biomass [18]. When improperly managed, such waste can lead to environmental pollution or the transmission of diseases [5]. However, valorizing agro-waste into high-value products represents a sustainable bioeconomic opportunity, offering cleaner, economically viable and socially beneficial supply chains while enhancing waste management systems [18]. Studies have demonstrated the potential of agro-waste-derived dyes in textiles. For instance, peanut skins [4], pomegranate rind [19], chickpea husks [5] and tannin extract from tamarind seed coat [20] are used to dye cotton fabrics [4].

Cotton, a widely used natural fibre, is a key substrate in the textile industry, and its hydrophilic nature and high affinity for various dyes make it an ideal candidate for natural dye applications [21]. However, achieving satisfactory dye adsorption, fastness and shade reproducibility remains challenging [3,22]. To address these issues, researchers have developed techniques to enhance dye–fibre interactions. Mordants, in particular, play a crucial role in improving dye fixation, fastness and colour. Innovative methods like pre-treatment of fibres and enzymatic processing are also being explored to further improve the dyeing process and enhance the overall sustainability of cotton dyeing [3].

The dyeing mechanism of natural dyes on cotton cellulose primarily involves non-covalent interactions between dye chromophores and the abundant hydroxyl groups present in the cellulose structure. These interactions encompass hydrogen bonding and van der Waals forces, which collectively determine the dye uptake and colour fastness properties [23]. The negatively charged cellulose surface under alkaline conditions creates electrostatic repulsion with anionic dye molecules, necessitating the use of auxiliary chemicals or fibre modifications to enhance dye–fibre affinity [24]. When mordants are employed, polyvalent metal ions (such as Al^3+^, Fe^3+^, Cu^2+^) or bio-mordants serve as coordination bridges, forming stable ternary dye–mordant–fibre complexes that significantly improve both dye substantivity and colourfastness through chelation and coordination bonding mechanisms [23].

To overcome the inherent limitations of natural dyes on cotton, multiple innovative strategies have been extensively investigated to improve dye uptake, colour depth and durability [25]. Cationization represents a groundbreaking approach that involves the chemical modification of cellulose by introducing permanent positive charges through quaternary ammonium groups, typically using reagents such as 3-chloro-2-hydroxypropyltrimethylammonium chloride or glycidyltrimethylammonium chloride [26]. This modification fundamentally alters the fibre’s surface charge, promoting stronger electrostatic attraction with anionic dye molecules and enabling complete elimination of inorganic salts from the dyeing process [27], thereby addressing the environmental concerns associated with conventional reactive dyeing while achieving enhanced colour yields and levelness [26]. Mordanting strategies have evolved beyond traditional metallic mordants to incorporate environmentally benign alternatives, including bio-based mordants such as tannic acid, chitosan and protein-based compounds, which facilitate the formation of coordination complexes between natural dyes and cellulose hydroxyl groups while minimizing environmental impact and maintaining excellent wash fastness properties [23,25,28]. Plasma treatment has emerged as a clean and efficient surface modification technique that significantly enhances fibre wettability and introduces reactive functional groups on the cotton surface through controlled oxidation processes, resulting in improved dye penetration, uniformity and overall dyeing performance without the use of harsh chemicals [29]. The plasma activation process increases surface energy and creates reactive sites that facilitate better dye–fibre interactions, particularly when combined with subsequent chemical modifications [29]. Biopolymer-assisted dyeing, particularly utilizing chitosan as a cationic biopolymer, has demonstrated remarkable efficacy in natural dyeing applications [28], where the protonated amino groups of chitosan under acidic conditions provide additional binding sites for anionic dyes while simultaneously imparting antimicrobial properties to the treated fabrics, thus offering a multifunctional approach to textile enhancement that aligns with sustainable manufacturing principles [23,26].

**Figure 1.**
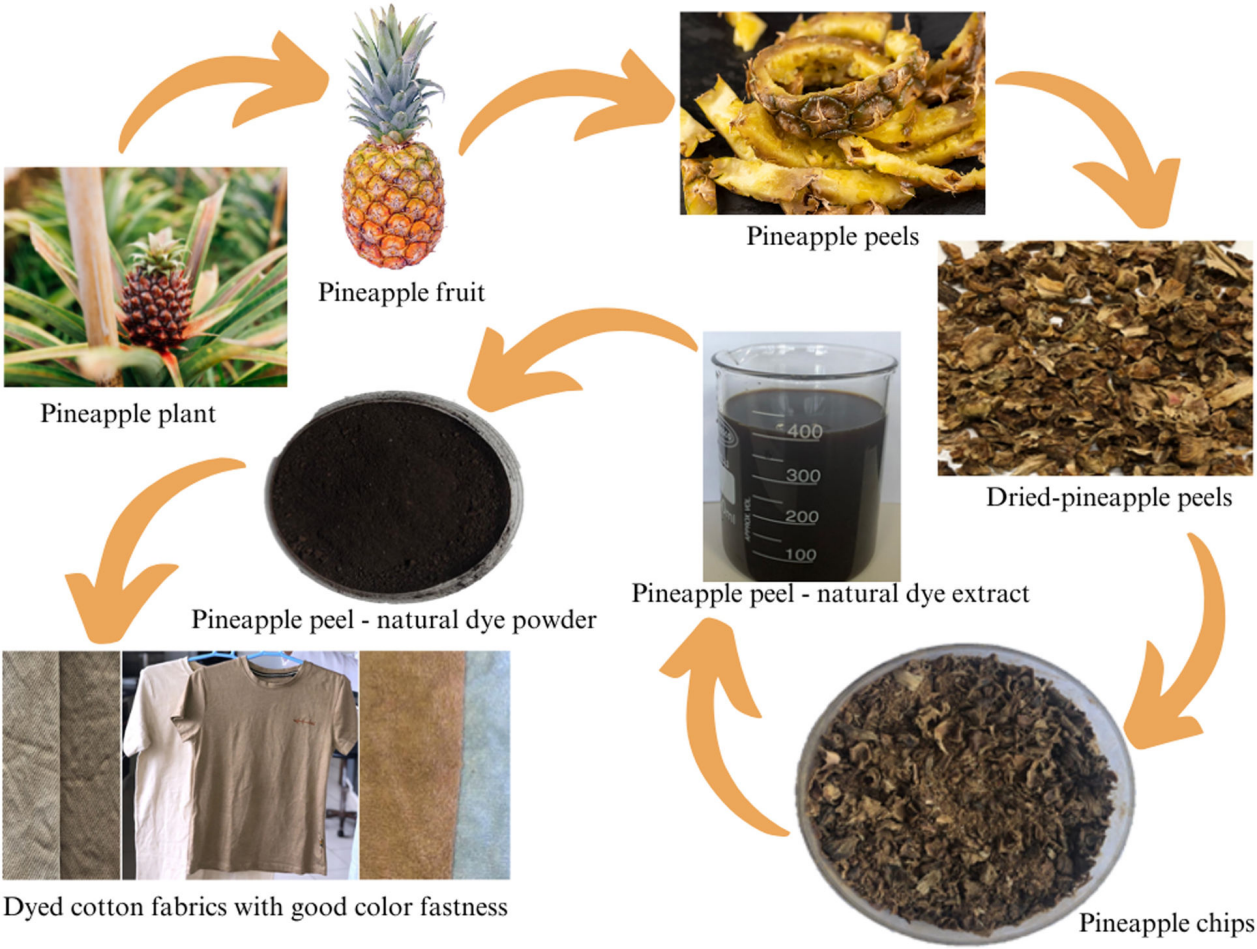
Pineapple peel-derived natural dye extraction process.

**Figure 2.**
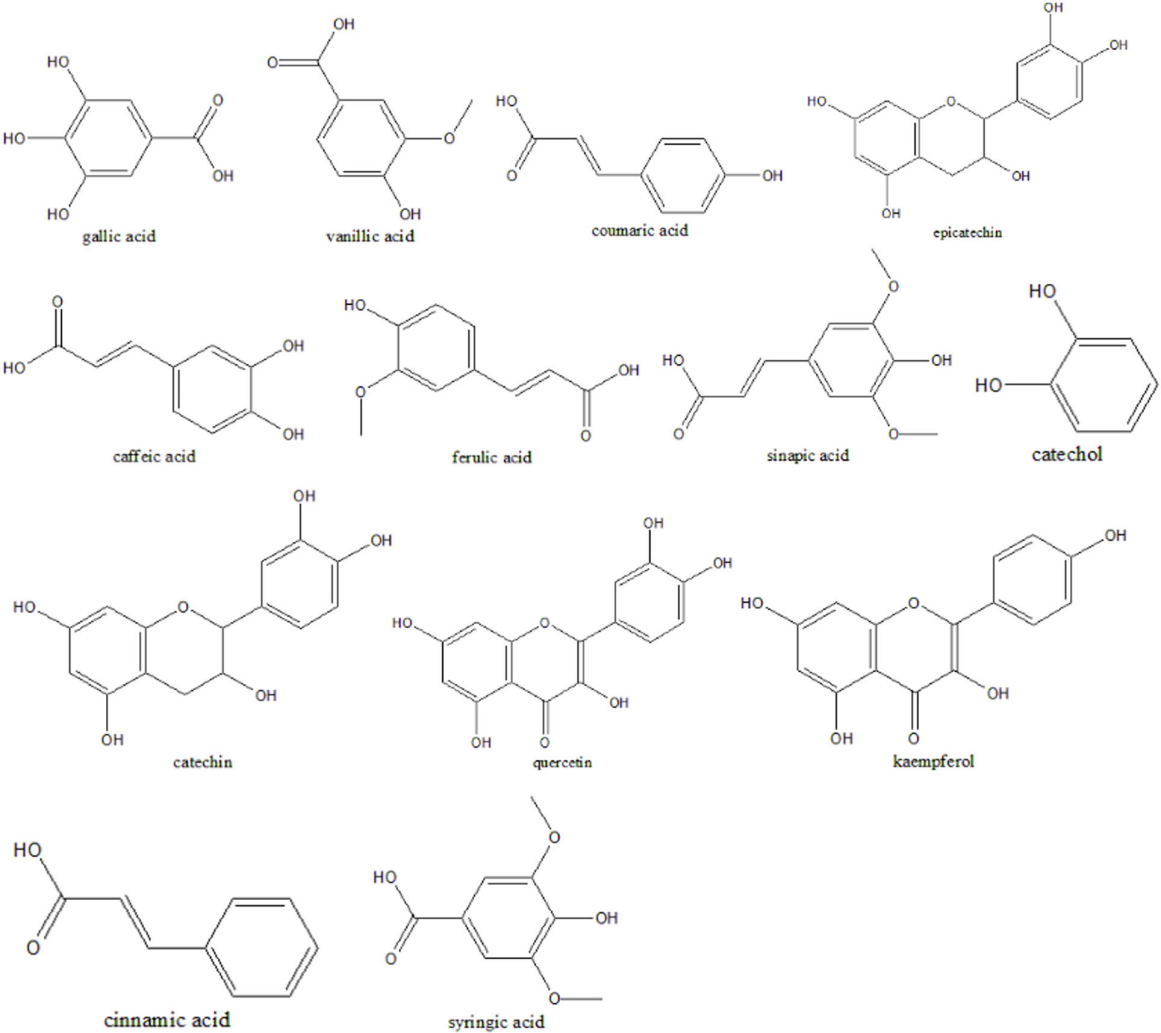
Major chemical constituents of pineapple peel [25].

An innovative method for sustainable textile dyeing is explored in this study, utilizing pineapple peel waste (figure 1) as a natural colourant. Pineapple peel, a significant agro-waste, offers a rich source of bioactive compounds (figure 2) such as polyphenols and flavonoids, which can be harnessed to produce eco-friendly dyes for textiles and other applications [30,31]. Pineapple is widely cultivated in tropical and subtropical regions, which collectively produce millions of tons of pineapple annually [32]. The peels, constituting a substantial portion of the fruit, are often discarded as waste. In Sri Lanka, pineapple (*Ananas comosus*) plays a significant role in agriculture, with its cultivation satisfying both local consumption and export markets, particularly in the form of pineapple juice. Sri Lanka produces two commercially cultivated pineapple types [33]. The Mauritius variety provides relatively smaller fruits, whereas the Kew variety provides large fruits that are primarily used for canning. The Mauritius variety is commonly farmed in Sri Lanka. Pineapple cultivation in Sri Lanka thrives in regions with favourable climate and soil conditions, supporting extensive production across various districts [34]. Key pineapple export markets include the Maldives, Germany and The Netherlands, which together account for a significant portion of Sri Lanka’s pineapple exports, where Sri Lankan pineapples are exported in various forms such as juice, dried and fresh [35].

Pineapple processing often results in the discarding of its peel as waste. The disposal of pineapple peel waste has become an increasingly concerning issue due to its susceptibility to microbial spoilage, leading to serious environmental consequences [18]. Pineapple peel waste produces greenhouse gases such as methane when it degrades in landfills, which can be harmful to the environment if not managed properly [33]. Proper management of pineapple waste can have significant benefits, aligning with the United Nations Sustainable Development Goals and promoting responsible consumption and production. This can reduce the environmental impact of textile dyeing and result in zero carbon dioxide emissions while generating valuable products [18].

The novel strategy of the present study lies in developing a reproducible, sustainable dyeing approach using powdered pineapple peel waste extract. This involves alkaline aqueous extraction of colourant, optimization of extraction parameters, powder conversion for storage stability and optimization of mordant and cationizing treatments to enhance dye performance on cotton fabric. Unlike previous research, this study systematically examines the effects of pH, temperature and mordant concentration on dye uptake and fastness. The colorimetric properties of the dyed cotton are assessed to identify optimal dyeing conditions, and the results are interpreted in terms of both functional and environmental impact. By establishing a replicable framework for utilizing pineapple peel dye powder in textile applications, this work contributes to eco-friendly, value-added solutions for the dyeing industry.

## 2. Experimental details

### 2.1. Chemicals and reagents

All chemicals and reagents used in this study were of analytical grade and employed without further purification. Methanol (CH_3_OH; CAS number: 67-56-1), hexane (CH_3_(CH_2_)_4_CH_3_; CAS number: 110-54-3), acetic acid (CH_3_COOH; CAS number: 64-19-7), ethanol (C_2_H_5_OH; CAS number: 64-17-5) and sodium chloride (NaCl; CAS number: 7647-14-5) were laboratory-grade reagents. Sodium hydroxide (NaOH; CAS number: 1310-73-2), potassium permanganate (KMnO_4_; CAS number: 7722-64-7), zinc sulfate (ZnSO_4_; CAS number: 7733-02-0), aluminium sulfate (Al_2_(SO_4_)_3_; CAS number: 10043-01-3), ammonium ferrous sulfate (AFS) (Fe(NH_4_)_2_(SO_4_)_2_.6H_2_O; CAS number: 7783-85-9), and tannic acid (C_76_H_52_O_46_; CAS number: 1401-55-4) were obtained from Sigma Aldrich, MO, USA, while the cationizer Besol OED (commercial quaternary ammonium-based product), bio-polish ndx, fixing agent Novafix (cationic), direct dye (zeta direct), Sumifix blue E-XF, and Sumifix red E-XF were sourced from S & D Chemicals (Pvt) Ltd.

### 2.2. Sample collection and preparation

Pineapple (*A. comosus*) peel waste was sourced from a fruit export company based in Colombo, Sri Lanka. The collected pineapple peel was thoroughly washed to remove impurities, followed by dehydrating at 160°C for 1 h using an oven to ensure proper drying. The dried peel was ground into fine chips to facilitate subsequent processing steps.

### 2.3. Extraction and optimization of colourants from pineapple peel

#### 2.3.1. Effect of extraction medium for colourant extraction

To extract colourants, finely ground pineapple peel chips were suspended in distilled water and subjected to heating at 60°C with magnetic stirring for 1 h to facilitate colourant release. The resulting solution was filtered twice to ensure clarity. The extraction process was repeated using various extraction mediums as a comparative solvent screening across a polarity range, including organic solvents (hexane, ethanol and methanol) and water-based systems such as alkaline solutions (water/NaOH), acidic solutions (water and acetic acid) and water with added inorganic salts. All extractions were performed under identical heating, stirring and filtration conditions. Through this comparative analysis, the most efficient extraction medium for pigment extraction was identified, ensuring maximum colourant yield.

#### 2.3.2. Optimization of alkaline extraction parameters

A water/NaOH extraction medium was employed to optimize the extraction of pigments under varying conditions of time (1 h, 1.5 h, 2 h, 2.5 h, 3 h), temperature (40°C, 50°C, 60°C, 70°C, 80°C), material-to-liquid ratio (MLR) (1 : 10, 1 : 15, 1 : 20, 1 : 30) and alkaline concentration (5%, 8%, 10%, 12%). During the optimization experiments, each parameter was varied independently, keeping all other conditions constant, and then the resulting extracts were subjected to centrifugation to remove residues at 3500 r.p.m. for 20 min, and the solutions were subsequently diluted 100-fold. The absorbance of the extracts was measured using a UV–visible (UV–vis) spectrophotometer to assess pigment concentration and extraction efficiency.

##### 2.3.2.1. Experimental design and optimization

A four-factor linear regression model was employed to investigate the individual and combined effects of extraction parameters on the dye yield. The independent variables studied were MLR (*X*_1_), NaOH concentration (*X*_2_), extraction temperature (*X*_3_) and extraction time (*X*_4_). The experimental ranges for each factor were selected based on preliminary studies: MLR varied from 0.03 to 0.1, NaOH concentration from 0.05 to 0.12, temperature from 40 to 80°C, and extraction time from 1 to 3 h. The extraction efficiency was quantified by measuring the absorbance of the resulting extract, which serves as an indicator of the concentration of extracted chromophores.

The experimental data were analysed using linear regression to determine the significance of individual factors and their effects on extraction efficiency. The mathematical model relating the response variable absorbance (*Y*) to the independent variables was expressed as equation (2.1):


(2.1) $$Y=\;\beta_{0}+\beta_{1}X_{1}+\beta_{2}X_{2}+\beta_{3}X_{3}+\beta_{4}X_{4},$$

where *β*_0_ represents the intercept and *β*_1_, *β*_2_, *β*_3_ and *β*_4_ are the regression coefficients for MLR, NaOH concentration, temperature and time, respectively. Statistical analysis was performed using Jamovi 2.6.44 software with significance evaluated at *α* = 0.05. Analysis of variance (ANOVA) was conducted to assess the overall model significance and the individual contribution of each factor. Model adequacy was evaluated through examination of residuals, coefficient of determination (*R*^2^), and other diagnostic measures to ensure the validity of statistical conclusions.

### 2.3.3. Extraction of colourants using optimized parameters

The optimal extraction conditions were achieved with an MLR of 1 : 10 in a water/12% NaOH extraction medium. The mixture was heated to 80°C and maintained for 2 h, effectively solubilizing the colourants. After filtration to remove solid residues, the resulting liquid extract was either used directly as a textile dye or further processed into a powder form via oven drying at 160°C. To stabilize the pH and inhibit fermentation, 10 g l^−1^ of NaCl was added to the filtered extract. The yield, representing the proportion of dried dye powder obtained from the initial dry weight of pineapple peel chips, was calculated following the optimized dye extraction process.

#### 2.3.3.1. High-performance liquid chromatography analysis

The polyphenolic compounds present in the water and alkaline (NaOH) extracts of pineapple peel were analysed using a C18 column with an Agilent LCMS fitted with a 1100/1200 photodiode array detector, 1100/1200 quaternary pump and 1100 auto sampler. The mobile phase consisted of a binary solvent mixture of 0.1% acetic acid solution (solvent A) and acetonitrile (solvent B) operated in linear gradient mode. The gradient programme was optimized as follows: initial phase maintained at 100% solvent A for 0 min, followed by a gradient from 100% A to 70% A over 40 min, then from 70% A to 40% A over 20 min, subsequently from 40% A to 10% A over 2 min, held at 10% A for 6 min, returned to initial conditions over 2 min, and equilibrated at initial conditions for 5 min, resulting in a total run time of 75 min. The mobile phase pH was maintained in the acidic range (2.5–3.0). The flow rate was set at 1 ml min^−1^, and the column temperature was maintained at room temperature (25°C). Sample injection volume was 10 µl, and the compounds were monitored using the photodiode array detector with wavelengths ranging from 210 to 500 nm, with specific monitoring at 280 nm for phenolic compound detection. All samples were filtered through 0.45 µm PTFE filters prior to injection to remove particulate matter.

### 2.3.4. Pre-treatment of cotton fabric

**Figure 3.**
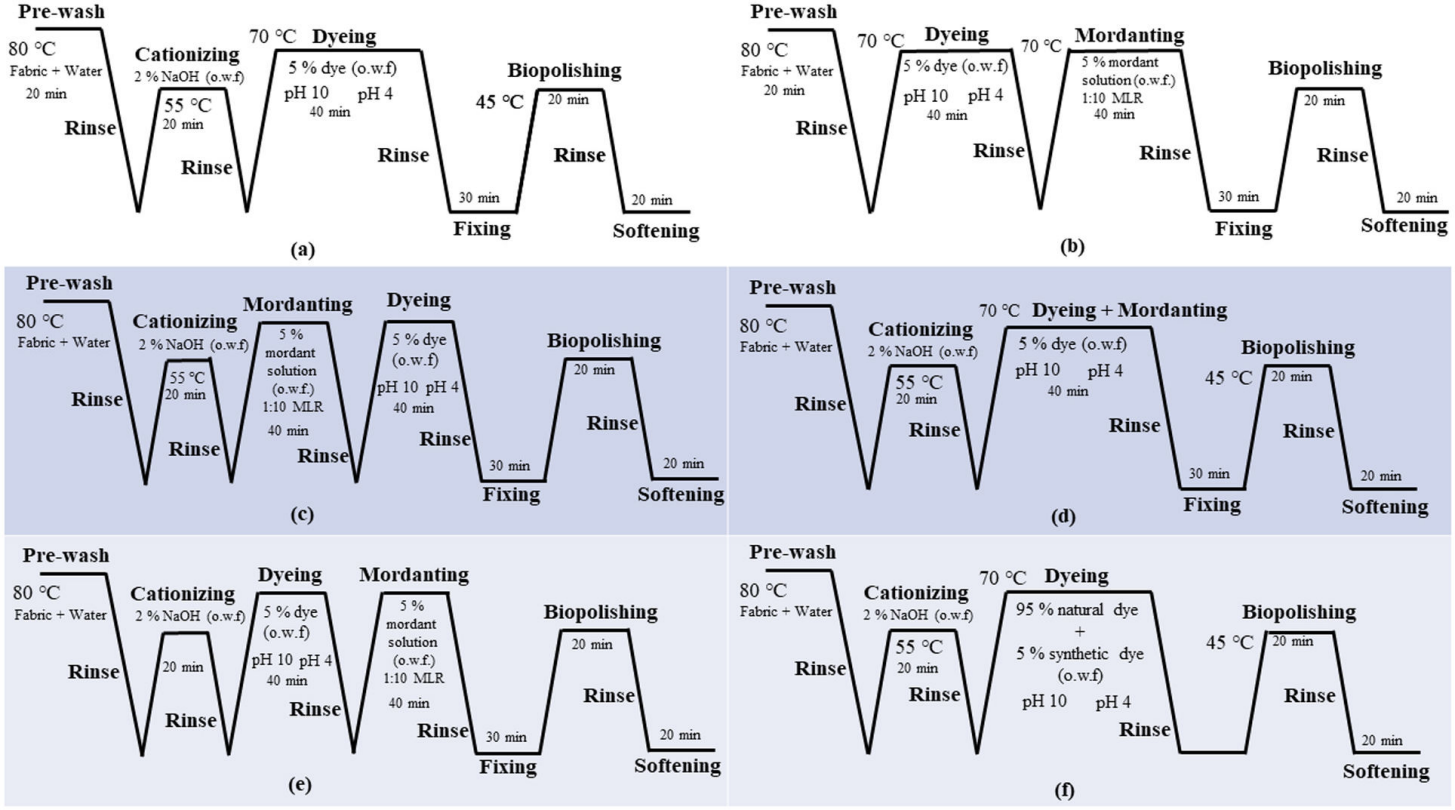
Schematic diagram of dyeing profile: (a) dyeing without mordanting, (b) dyeing without cationizing, (c) pre-mordanting, (d) simultaneous mordanting, (e) post-mordanting and (f) dyeing with a blend of natural and synthetic dyes (95% : 5%).

Cotton fabric underwent a pre-treatment process to improve dye absorption. A cationizing agent—Besol OED (10 g l^−1^)—combined with an MLR of 1 : 10, 2% NaOH on weight of fabric (o.w.f) was applied to the fabric at 50–60°C for 20 min. After the treatment, the fabric was thoroughly rinsed with water. Three mordanting techniques—pre-mordanting, simultaneous mordanting and post-mordanting—were applied (figure 3). In pre-mordanting, the fabric was treated in 5% (o.w.f) mordant solutions (aluminium sulfate, zinc sulfate, ammonium ferrous sulfate and tannic acid) at 70°C for 40 min with an MLR of 1 : 10, followed by rinsing. Simultaneous mordanting involved treating the fabric in dye baths containing both pineapple peel (PA) dye and mordants under the same conditions. Post-mordanting was performed after the initial dyeing, by treating the fabric in the same mordant solutions, followed by rinsing and drying.

### 2.3.5. Dyeing methods and conditions

Dyeing parameters such as time, temperature and pH were studied. The fabric was dyed using PA dye powder equivalent to 5% of the fabric’s weight. It was immersed in the dye solution and heated at 70°C for 20 min at pH 10, followed by an additional 20 min at pH 4. Adjustment of pH was done using acetic acid. The MLR was maintained at 1 : 10, with continuous stirring to ensure uniform dye distribution. After dyeing, the fabric was thoroughly rinsed with water, squeezed to remove excess liquid and dried completely. Fixing agents—Novafix—were applied to improve colour fastness properties, followed by biopolishing agents to enhance surface smoothness and texture. A final rinse was performed, and softening agents were applied to improve the fabric’s hand feel.

To broaden the colour spectrum, a dye mixture was prepared keeping the total dye concentration of 5% (w/w), combining 95% PA dye and 5% synthetic dyes. Two types of synthetic dyes, direct and reactive, were evaluated for compatibility and levelness when combined with PA dye. The dyeing process for these mixtures was conducted under the optimized conditions detailed above with the addition of PA dye, the only modification being the incorporation of the combination of natural and synthetic dye. Visual assessment was performed on the dyed samples to evaluate colour levelness. To further enhance the colour palette, 1% (w/w) potassium permanganate (KMnO_4_) was applied to the PA-dyed fabric (table 1).

**Table 1.** Experimental protocol for PA dyeing of cotton fabric: complete processing conditions from pre-treatment to finishing.

process stage	parameter	condition
pre-treatment	cationizing agent	Besol OED (10 g l^−1^)
	cationization conditions	50–60°C, 20 min, MLR 1 : 10
	alkali addition	2% NaOH (o.w.f.)
	post-treatment	thorough water rinsing
mordanting	mordant types	aluminium sulfate, zinc sulfate, AFS, tannic acid
	mordant concentration	5% o.w.f.
	mordanting conditions	70°C, 40 min, MLR 1 : 10
	mordanting methods	pre-, simultaneous, post-mordanting
dyeing	dye source	PA dye powder
	dye concentration	5% o.w.f.
	dyeing temperature	70°C
	dyeing time	20 min at pH 10 and 20 min at pH 4
	pH adjustment	acetic acid
	MLR	1 : 10
	post-dyeing	water rinsing, squeezing and complete drying
after treatment	tannic acid treatment (mordant)	10% o.w.f., 50°C, 20 min, MLR 1 : 10
	dye-fixing agent	Novafix (cationic)—2 g l^−1^ at 45°C
	softening agent	cationic softener—1 g l^−1^ at room temperature
	finishing treatment	biopolishing and final rinse
dye mixtures	synthetic dye blends	95% PA dye + 5% synthetic (direct/reactive)
	total concentration	5% (w/w)

### 2.3.6. After-treatment of the dyed cotton fabric

To improve the colour fastness of the dyed cotton fabric, tannic acid (a plant-derived polyphenol) was applied at 10% o.w.f. as one of the mordants. The fabric was treated at an MLR of 1 : 10 for 50°C and 20 min. Following this, additional after-treatment steps were performed to further enhance the fabric’s properties, including colour fastness, durability, softness and resistance to colour bleeding. This included treating the fabric with 2 g l^−1^ of Novafix (a cationic dye-fixing agent), at 45°C to improve dye retention and ensure colour stability. Afterwards, a cationic softener was added at a concentration of 1 g l^−1^ at room temperature to improve the fabric’s softness and feel. These after-treatment steps, including the application of finishing agents, surface modifiers and fixing agents as per the dyeing profile, ensured enhanced colour fastness, durability and overall fabric quality.

### 2.3.7. Characterization of the prepared dye powder

The thermal stability of the extracted dye powder was determined using an SDT Q600 thermogravimetric analyser within a temperature range of 27–1000°C, with a sample mass of 12.80 mg. Particle size analysis was conducted using a Malvern Zetasizer Nano ZS, where 0.1 g of dye powder sample was dissolved in 100 ml of distilled water to prepare the sample. Fourier-transform infrared (FTIR) spectroscopy was utilized to identify the functional groups present in the raw material, the extracted dye and the dyed fabrics. The FTIR analysis included samples treated with various mordants and dyeing techniques to assess the chemical interactions and modifications resulting from the dyeing processes.

### 2.3.8. Dye exhaustion and fixation analysis

The dyeing efficiency was quantitatively evaluated through spectrophotometric determination of dye exhaustion, total fixation and fixation ratio. The extent of the dye exhaustion was determined by measuring the absorbance of the dye bath solution before and after the dyeing process at the *λ*_max_ of the PA dye. The percentage dye bath exhaustion (%*E*) was calculated using equation (2.2):


(2.2) $$\%E=\;\left[\frac{\left(A_{0}-A_{1}\right)}{A_{0}}\right]\times 100\%,$$

where *A*_0_ and *A*_1_ are the absorbance values of the dye bath before and after dyeing, respectively.

The total fixation efficiency, representing the percentage of dye permanently fixed to the cotton fibre, was determined spectrophotometrically. Following the dyeing process, dyed fabric samples underwent a systematic wash-off procedure to remove unfixed dye. The samples were first rinsed in cold running water until no further colour desorption occurred, then boiled in distilled water for 15 min at 100°C, followed by a final rinse in cold running water until no additional dye was removed. Wash-off solutions from these steps were collected for analysis. The total fixation efficiency was calculated using equation (2.3):


(2.3) $$\%T=\;\left[\frac{\left(A_{0}-A_{1}-A_{2}\right)}{A_{0}}\right]\times 100\%,$$

where *A*_0_ is the absorbance of the original dye bath before dyeing, *A*_1_ is the absorbance of the dye bath after dyeing and *A*_2_ is the absorbance of the wash-off solutions. All absorbance measurements were performed using a UV–vis spectrophotometer at the characteristic absorption maximum of the PA dye (280 nm).

From the dye exhaustion and total fixation efficiency values, the fixation ratio of the absorbed dye (%*F*) was calculated using equation (2.4):


(2.4) $$\%F=\frac{\%T}{\%E}.$$

The fixation ratio represents the proportion of exhausted dye that is actually fixed to the fibre, providing insight into the binding strength between the dye and cotton substrate.

### 2.3.9. Colour measurement

Colour measurement of the dyed fabric samples was performed using a Datacolor spectro 700 benchtop spectrophotometer under standard illuminant conditions. The colour values were determined based on coordinates in the CIELab colour space system, where L* represents lightness/darkness with values measured relative to undyed white cotton fabric as the control (dL* = *L*_sample_ – *L*_white cotton_) ranging from 0 (black) to 100 (white), a* represents the red–green axis with positive values indicating red and negative values indicating green, and b* represents the yellow–blue axis with positive values indicating yellow and negative values indicating blue [36]. The measurements were conducted using D65/10° measurement geometry with msTL84-10/A/10 standard and DEcmc tolerance settings with *L* : *c* ratio of 2.00 : 1.00.

Colour strength analysis of the fabric samples was performed using a Shimadzu UV-3600 UV–vis–NIR spectrophotometer in the wavelength range of 400–700 nm. The reflectance values (%*R*) were recorded for each sample, and the colour strength (*K*/*S*) values were calculated from the reflectance data using the Kubelka–Munk equation (2.5):


(2.5) $$\frac{K}{S}=\;\frac{{\left(1-R\;\right)}^{2}}{2R},$$

where *K* is the absorption coefficient, *S* is the scattering coefficient and *R* is the reflectance value in decimal form.

Statistical analysis was performed using Jamovi 2.6.44 software. Descriptive statistics, including mean, s.d., variance, minimum and maximum values, were calculated for all colour parameters (L*, a*, b*, *K*/*S*) across different mordant types. One-way ANOVA (Welch’s test) was conducted to evaluate significant differences in colour strength (*K*/*S*) values between mordant types, with homogeneity of variances assessed using Levene’s test. *Post hoc* analysis was performed using the Games–Howell test for pairwise comparisons between mordant groups. Correlation analysis was conducted using Pearson’s correlation coefficient to determine relationships between colour parameters. Statistical significance was set up at *p* < 0.05.

### 2.3.10. Colour fastness properties

After optimizing the dyeing conditions, colour fastness tests were conducted to evaluate the durability of the dye under various conditions. The colour fastness to light was tested using a water-cooled xenon arc lamp adhering to the American Association of Textile Chemists and Colorists (AATCC) TM16.3-2021, which evaluates fading properties under control conditions. For perspiration resistance, AATCC TM15-2021 was employed to assess the fabric’s performance under simulated sweat conditions. The colour fastness to laundering was tested using AATCC TM61-2021, involving accelerated laundering at 49°C with AATCC standard powder detergents. Changes in colour and staining levels were evaluated using the grey scale, following AATCC Evaluation Procedure 2-2021. Rub fastness tests were performed to evaluate the resistance of the dyed fabric to surface abrasion in both dry and wet conditions, adhering to AATCC TM8-2021 standards. The outcomes of all fastness evaluations are summarized in table 2.

**Table 2.** Fastness properties of natural and hybrid dyes on cotton fabric under various mordanting conditions, including light, laundering, perspiration (acidic and alkaline) and rubbing (dry and wet).

dye source/method	sample	colour fastness						
		light	water	laundering	perspiration		rubbing	
					acid	alkaline	dry	wet
pineapple peel dye (only)	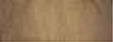	1	4	3	3	2–3	3	2
pineapple peel dye + KMnO_4_	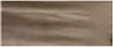	2–3	4	3–4	2–3	2–3	4	4
Sumifix red E-XF	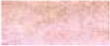	2–3	4	3	4	4	3–4	3
Sumifix blue E-XF	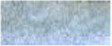	2–3	4	3	4	4	3–4	3
direct dye	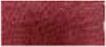	2–3	3–4	4	4	4	4	4
cationized (without mordanting)	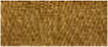	1–2	3	4	3–4	3–4	3	2–3
cationized + alum (post-mordanting)	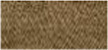	2	3–4	4	2–3	2–3	4	3
cationized + ammonium ferrous sulfate	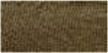	2–3	3–4	4	3	3	3	3
cationized + zinc sulfate (mordanting)	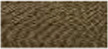	2	4–5	5	4	4	4	3
alum (pre-mordanting)	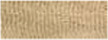	1	4	3	2–3	2–3	3	2–3
alum (simultaneous mordanting)	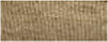	1	3–4	2–3	2	2	3	2
alum (post-mordanting)	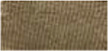	1	3	3	3	2–3	4	2–3
zinc sulfate (pre-mordanting)	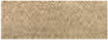	1–2	5	4–5	4	4	3-4	3
zinc sulfate (simultaneous mordanting)	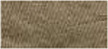	1	4	4	3–4	3–4	3	2–3
zinc sulfate (post-mordanting)	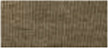	4	5	4–5	4	4	4	4
ammonium ferrous sulfate (pre-mordanting)	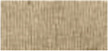	1	4	3	3	3	2–3	2–3
ammonium ferrous sulfate (simultaneous mordanting)	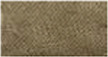	2–3	2–3	3	2–3	2–3	3	3
ammonium ferrous sulfate (post-mordanting)	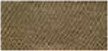	2–3	3–4	3–4	3	3	2	2–3
tannic acid (pre-mordanting)	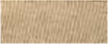	1–2	3	3	3–4	3–4	3	2–3
tannic acid (simultaneous mordanting)	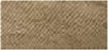	2	3	3	3	3	3–4	2–3
tannic acid (post-mordanting)	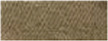	3	3–4	4	3–4	3–4	4	3–4

## 3. Results and discussion

### 3.1. Optimization of extraction conditions

To determine the optimal conditions for pigment extraction, various extraction media were tested using a Shimadzu UV-3600 UV–vis–NIR spectrophotometer together with UV Probe software, including hexane, ethanol, methanol and water-based solutions. The effectiveness of each solvent was evaluated based on the extraction yield. Among the tested systems, the water/NaOH solvent system demonstrated superior efficiency, yielding 35–40% pigment extraction, meaning that 100 g of dried pineapple peel chips produced approximately 35–40 g of dried dye powder compared to the other extraction mediums. This higher yield is attributed to the high pH, which causes polyphenols to deprotonate, imparting an overall anionic nature to the pigments and enhancing their solubility in the alkaline medium. In contrast, the alternative solvents (hexane, ethanol and methanol) produced yields of less than 1%, indicating their limited ability to solubilize the pigments under the tested conditions.

**Figure 4.**
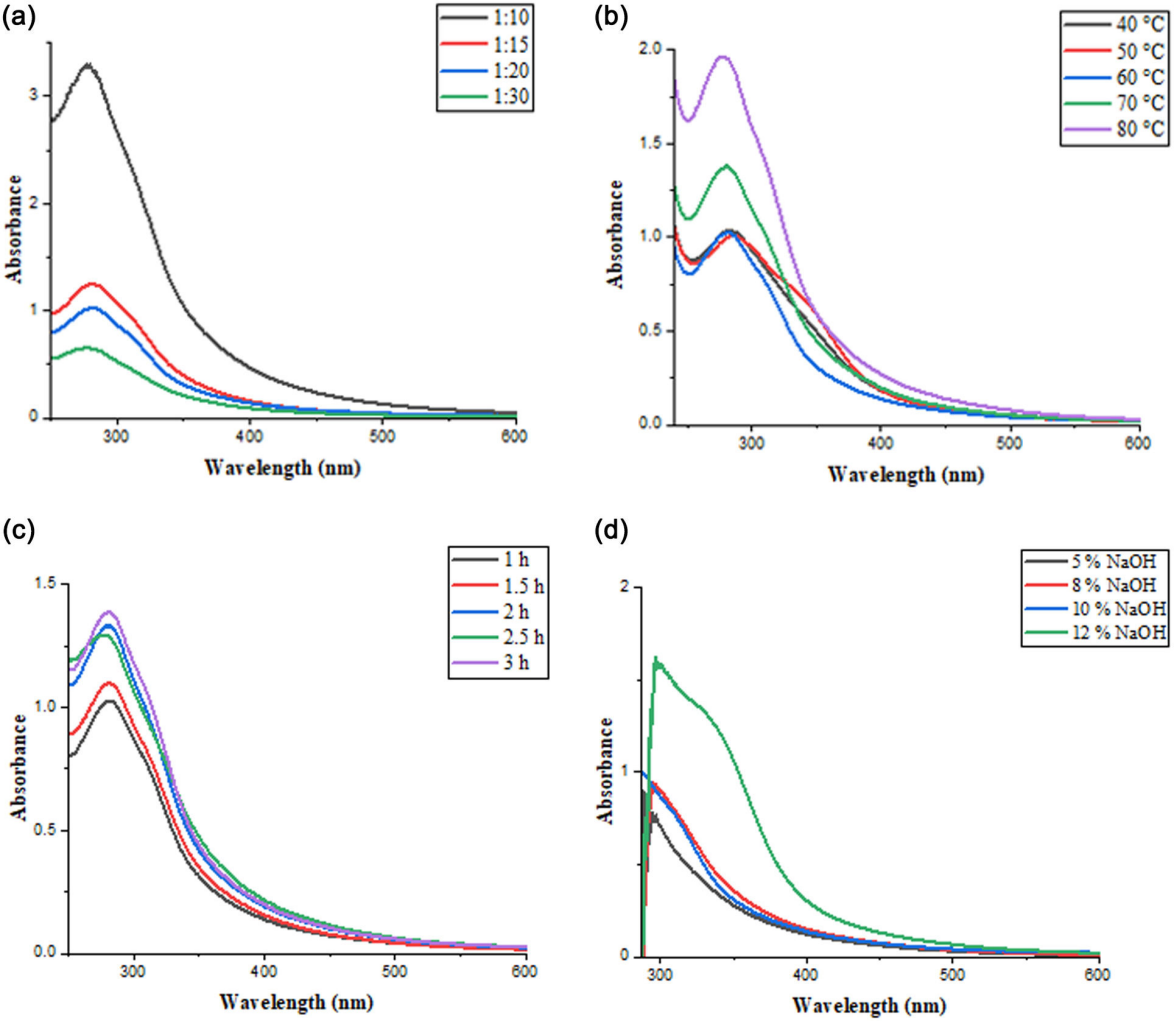
UV–vis spectra of dye extract solution showing: (a) effect of extraction with different MLR (1 : 10, 1 : 15, 1 : 20 and 1 : 30), (b) effect of extraction at different temperatures (40°C, 50°C, 60°C, 70°C, 80°C), (c) effect of extraction durations (1 h, 1.5 h, 2 h, 2.5 h, 3 h) and (d) effect of alkali concentration (5%, 8%, 10%, 12%).

The UV–vis spectroscopic data were analysed to optimize the extraction of natural colourants under varying experimental conditions. The parameters studied included MLR, NaOH concentration, temperature and extraction time (figure 4). The efficiency of colourant extraction was influenced by the choice of solvent and alkali concentration. The addition of NaOH aids in breaking down plant cell walls and tissues, facilitating the diffusion of colour components into the solvent. While the absorption spectra confirm the successful extraction of chromophoric compounds, the crude extract—comprising both colourants and other co-extracted organics—was utilized without further purification to simulate practical industrial dyeing practices, where minimal processing is preferred for cost and operational efficiency. The optimized extraction conditions, as shown in table 3, led to the maximum absorbance (280 nm) of the extracted pigments in the UV–vis spectra.

**Table 3.** Optimized extraction conditions.

parameter	optimum value
MLR	1 : 10
NaOH concentration	12%
temperature	80°C
time	2 h

The UV–vis spectra of the extracted pigments under varying conditions are presented in figure 4 where each graph illustrates the impact of a specific parameter on the absorption intensity, highlighting the optimal range for maximum extraction efficiency.

The optimized conditions resulted in a significant increase in absorbance at the characteristic wavelength of the extracted pigments, confirming the enhancement in pigment concentration. Among the tested MLRs, a lower MLR of 1 : 10 yielded the highest pigment concentration, due to reduced dilution and more contact between the solvent and the plant matrix. An alkali concentration of 12% effectively disrupted plant cell walls, promoting the release of colourant compounds. A temperature of 80°C was sufficient to promote the solubility of pigments without thermal degradation, and a 2 h extraction period allowed for maximum diffusion of colourants into the solvent (table 4).

**Table 4.** Complete experimental parameters for optimized colourant extraction from pineapple (*A. comosus*) peel and subsequent processing conditions.

parameter	condition
MLR	1 : 10
extraction medium	water/12% NaOH
temperature	80°C
extraction time	2 h
filtration	double filtration to remove solid residues, followed by centrifugation
drying method	oven drying at 160°C
final product forms	liquid extract or powder form
yield calculation	based on dried dye powder versus initial peel dry weight

#### 3.1.1. Statistical model performance

The extraction efficiency of natural dye was evaluated using a four-factor linear regression model to investigate the effects of MLR, NaOH concentration, temperature and extraction time on the absorbance values (table 5). The absorbance values correlate directly with dye yield. The linear regression analysis revealed that the four-factor model explained 31.5% of the variance in dye extraction efficiency (*R*^2^ = 0.315, Adjusted *R*^2^ = 0.104). The overall model showed moderate significance (*F* = 1.50, *p* = 0.260), indicating that while the selected factors contribute to extraction efficiency, additional variables may influence the process. Among the investigated parameters, NaOH concentration emerged as the most significant factor affecting dye extraction (*p* = 0.046, *F* = 4.885). The positive coefficient (*β* = 19.40) indicates that increasing alkaline conditions significantly enhances dye extract ion efficiency. This finding aligns with previous research demonstrating that alkaline conditions facilitate the breakdown of plant cell walls and improve the solubility of polyphenolic compounds [37]. MLR showed a moderate effect (*p* = 0.209) with a positive coefficient (*β* = 10.33), suggesting that optimizing the MLR ratio can improve extraction yield, though this effect was not statistically significant at the *α* = 0.05 level. Temperature (*p* = 0.303) and extraction time (*p* = 0.662) showed minimal influence on the extraction process under the tested conditions. The limited temperature effect in this study may be attributed to the specific extraction method employed or the nature of the target compounds. The residual analysis revealed some departures from normality (Shapiro–Wilk test, *p* = 0.004), suggesting that the linear model may not fully capture the complexity of the extraction process (table 6). The relatively low *R*^2^ value suggests that additional factors, such as particle size and pH optimization, may significantly influence the process. The dominance of NaOH concentration as the primary factor suggests that alkaline extraction conditions are critical for maximizing dye yield. This finding has practical implications for scale-up processes, where precise control of alkaline conditions would be essential.

**Table 5.** Experimental design matrix showing the independent variables and corresponding absorbance responses for PA dye extraction optimization.

	MLR	NaOH concentration	temperature (°C)	time (h)	absorbance at *λ*_max_
1	0.1	0.12	80	2	3.272
2	0.06	0.12	80	2	1.245
3	0.05	0.12	80	2	1.024
4	0.03	0.12	80	2	0.649
5	0.1	0.05	80	2	0.675
6	0.1	0.08	80	2	0.325
7	0.1	0.1	80	2	0.018
8	0.1	0.12	80	2	1.564
9	0.1	0.12	40	2	1.03
10	0.1	0.12	50	2	0.993
11	0.1	0.12	60	2	1.024
12	0.1	0.12	70	2	1.377
13	0.1	0.12	80	2	1.957
14	0.1	0.12	80	1	1.024
15	0.1	0.12	80	1.5	1.095
16	0.1	0.12	80	2	1.329
17	0.1	0.12	80	2.5	1.289
18	0.1	0.12	80	3	1.386

**Table 6.** Analysis of variance for the linear regression model evaluating the effects of extraction parameters on PA dye yield.

parameter	sum of squares	d.f.	mean square	*F*	*p*
MLR	0.7364	1	0.7364	1.747	0.209
NaOH concentration	2.0596	1	2.0596	4.885	0.046
temperature	0.4841	1	0.4841	1.148	0.303
time	0.0843	1	0.0843	0.200	0.662
residuals	5.4808	13	0.4216		

Note. Type 3 sum of squares.

#### 3.1.2. High-performance liquid chromatography analysis

**Figure 5.**
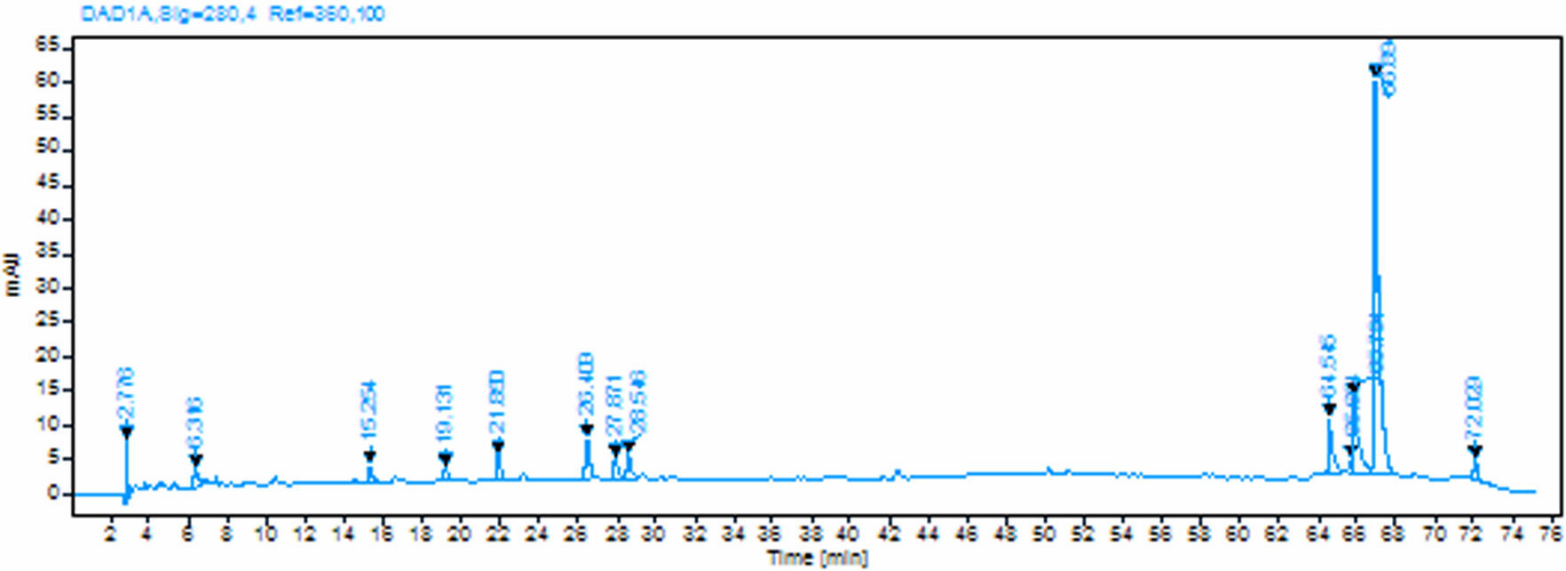
HPLC chromatogram of water extract of pineapple peel monitored at 280 nm.

The high-performance liquid chromatography (HPLC) analysis of pineapple peel extracts revealed distinct polyphenolic profiles between water and aqueous NaOH extraction methods. The chromatographic fingerprints demonstrated significant differences in both the number and intensity of detected compounds. The water extract of pineapple peel showed a complex polyphenolic profile with multiple peaks distributed throughout the chromatographic run (figure 5). At 280 nm detection wavelength, twelve distinct peaks were identified with retention times ranging from 2.776 to 72.029 min. The most prominent peak was observed at 66.894 min with the highest height (57.591 mAU), indicating the presence of a major polyphenolic component. Other significant peaks were detected at retention times of 65.794, 64.545 and 26.409 min.

**Figure 6.**
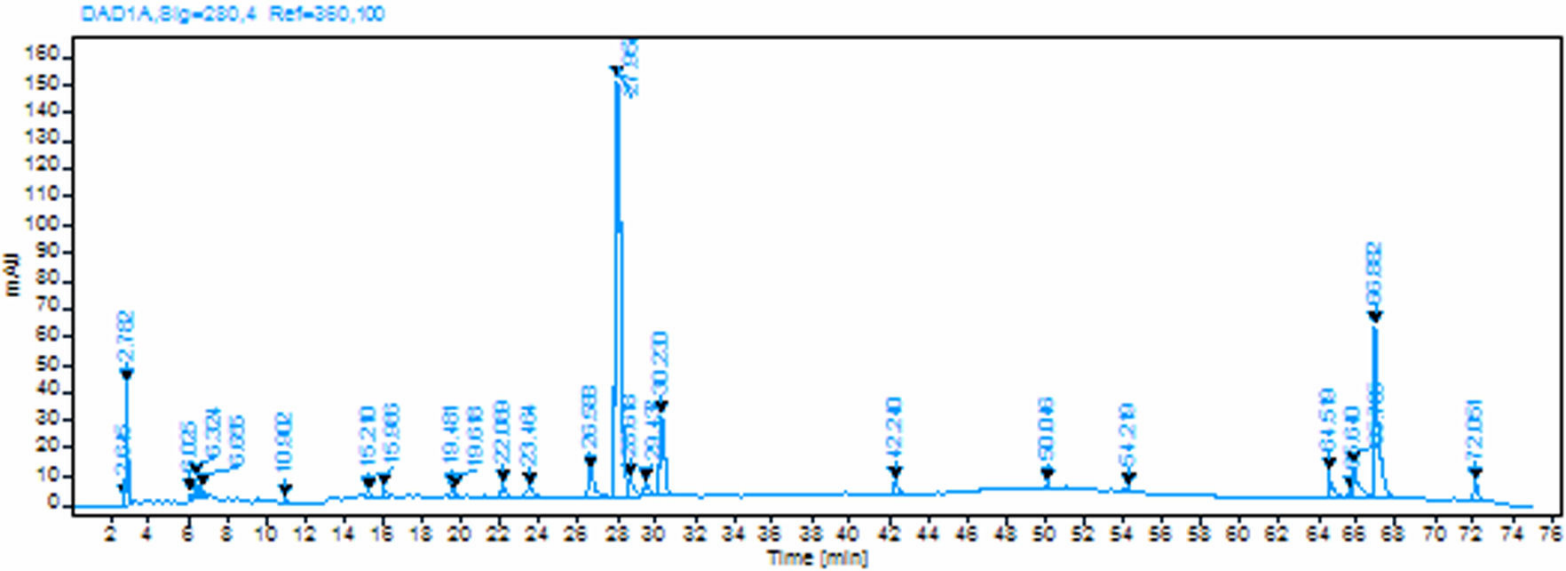
HPLC chromatogram of aqueous NaOH extract of pineapple peel monitored at 280 nm.

The aqueous NaOH extract demonstrated a markedly different chromatographic profile compared to the water extract, in terms of both peak distribution and relative intensities. At 280 nm detection, 26 peaks were identified across the chromatographic run, representing a significant increase in the number of detectable compounds compared to the water extract (figure 6). The dominant peak was observed at 27.957 min with an exceptionally high height (149.0509 mAU), indicating substantial extraction or conversion of polyphenolic compounds under alkaline conditions. Other notable peaks included those at 30.230, 66.882 and 26.588 min.

The comparative analysis between water and NaOH extracts revealed several important observations. Several major peaks present in the water extract, particularly those eluting at later retention times (>60 min), showed reduced intensity or complete disappearance in the NaOH extract. This phenomenon suggests that certain polyphenolic compounds undergo structural modifications, fragmentation, or degradation under alkaline conditions. Conversely, the alkaline extraction resulted in the emergence of numerous additional peaks, particularly in the early to mid-retention time range (15–35 min), indicating the release of previously bound or conjugated polyphenolic compounds, or the formation of new compounds through alkaline-induced transformations.

The shift in peak distribution from later retention times (characteristic of less polar compounds) to earlier retention times (more polar compounds) in NaOH extract suggests increased polarity of the extracted compounds, which is consistent with the deprotonation of phenolic hydroxyl groups under alkaline conditions. This polarity change enhances the solubility of certain polyphenolic compounds that may be poorly extracted in neutral aqueous conditions. The substantial increase in total peak area in the NaOH extract compared to the water extract indicates that alkaline conditions significantly enhance the extraction efficiency of polyphenolic compounds from the pineapple peel matrix. This enhancement may be attributed to several mechanisms, including: (i) breaking of hydrogen bonds and van der Waals interactions between polyphenols and cell wall components, (ii) hydrolysis of ester and glycosidic bonds, releasing bound phenolic acids and flavonoids, (iii) increased solubility due to phenolate ion formation, and (iv) potential oxidative polymerization or depolymerization reactions under alkaline conditions [3].

### 3.2. Optimization of dyeing parameters

The dyeing process was evaluated under three distinct pH conditions to determine the optimal conditions for achieving good dye fixation and colour depth. The conditions tested included: a gradual increase in pH from acidic (pH 4) to alkaline (pH 10), a gradual decrease in pH from alkaline (pH 10) to acidic (pH 4), and a neutral pH condition, maintained consistently at pH 7. Among these, the second condition, where the pH was gradually decreased from pH 10 to pH 4, provided the best dye depth and uniformity on the cotton fabric. Initially, alkaline conditions (pH 10) promoted partial ionization of hydroxyl groups in cellulose and deprotonation of phenolic hydroxyl groups in the dye, increasing their solubility and interaction potential. As the pH dropped, towards acidic conditions (pH 4), protonation of functional groups enhanced hydrogen bonding and dipole–dipole interactions between dye molecules and the cellulose matrix, leading to improved dye uptake. This gradual shift also helped minimize dye aggregation, promoting more uniform adsorption onto fibre surfaces. In contrast, the first condition (pH 4 to pH 10) and the neutral pH condition (pH 7) did not show satisfactory results exhibiting poor dye retention, leading to significant dye washout during rinsing, which resulted in inconsistent colouration and reduced dye depth, rendering these conditions less effective for achieving durable dyeing.

The effect of temperature on dyeing was studied under three conditions: non-cationized cotton, cationized cotton and mordanted cotton. On non-cationized cotton, dyeing at elevated temperatures (80–90°C) improved colour strength, likely due to enhanced fibre swelling and increased dye diffusion into the amorphous cellulose regions. However, due to the absence of ionic sites, dye retention was weak and mainly governed by non-covalent interactions. In cationized cotton, quaternary ammonium functional groups introduced via cationization imparted positive charges to the cellulose backbone. These sites electrostatically attracted the negatively charged dye molecules, allowing dyeing to proceed efficiently even at lower temperatures (below 50°C). This resulted in enhanced dye exhaustion, reduced energy consumption, and better shade uniformity.

**Figure 7.**
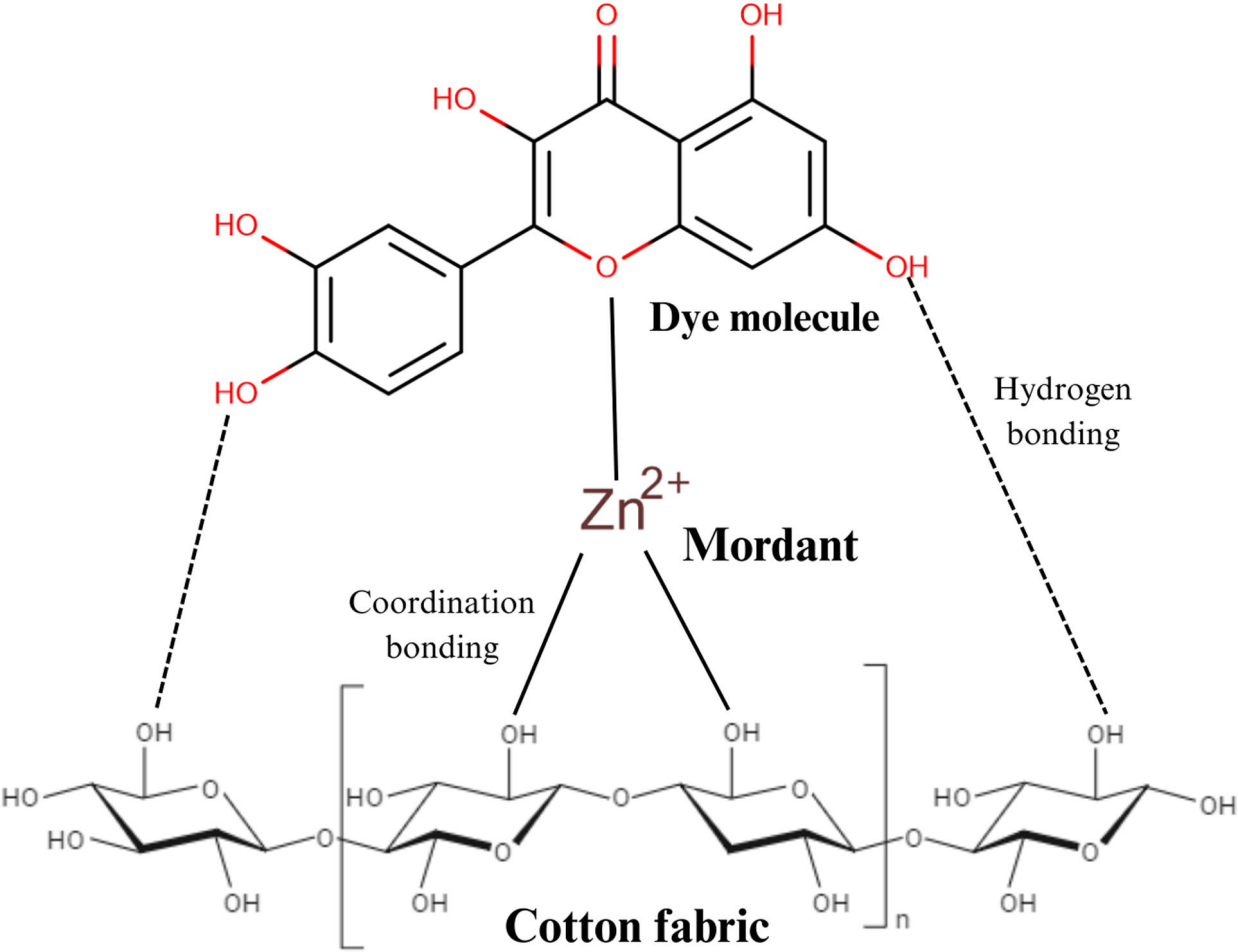
Schematic illustration of chemical interactions between the dye molecule and cotton fibre in the presence of Zn²^+^ as a mordant.

In mordanted cotton, metal salts (FeSO_4_, Al_2_(SO_4_)_3_, ZnSO_4_) were used to form coordination complexes with dye molecules. These metal–dye complexes could then interact with hydroxyl groups on cellulose via secondary bonding mechanisms (figure 7). Temperature optimization (70–85°C) was critical to prevent premature dye–metal complex formation in solution, which can lead to uneven dyeing. Among the three, cationized cotton outperformed as the most consistent and high-intensity colouration, owing to strong electrostatic dye–fibre interactions, supporting its suitability for energy-efficient and sustainable dyeing.

### 3.3. Compatibility of synthetic and pineapple peel dyes in cotton fabric dyeing

**Figure 8.**
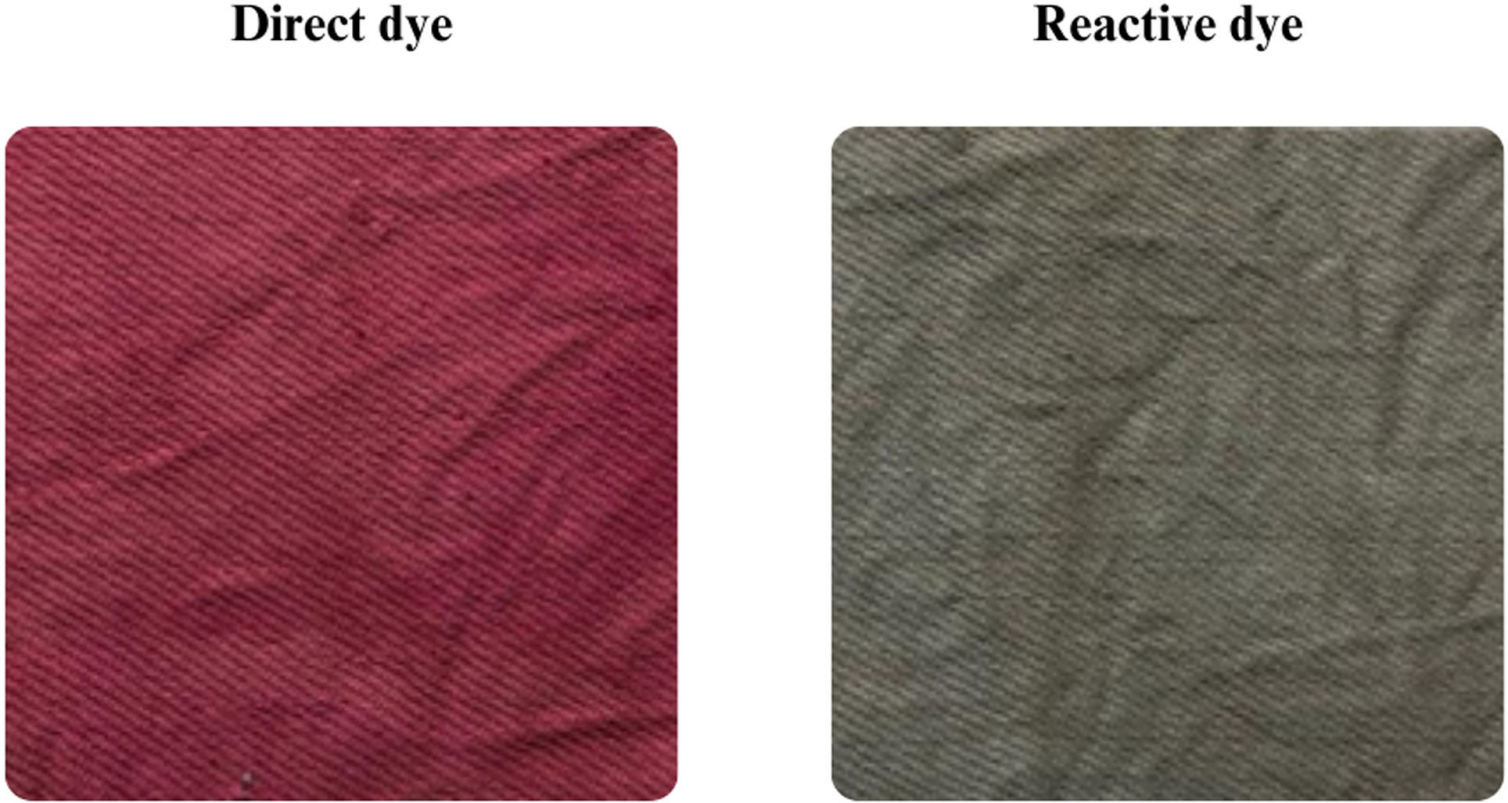
Colour uniformity and intermediate shades observed in synthetic dye blends mixed with PA dye, highlighting the performance of direct dye and reactive dye.

The study investigated the compatibility and dyeing performance of synthetic and PA dye blends on cotton fabrics. A total dye concentration of 5% (w/w) relative to the fabric weight was used, with each blend consisting of 5% synthetic dye (direct or reactive) and 95% PA dye. The resulting shade uniformity and colour distribution of the dyed fabrics were visually assessed. The blended colour outcomes were influenced by the hue of each synthetic dye (direct dye: red; reactive dye: black), producing intermediate shades when mixed with PA dye (figure 8).

Conversely, direct and reactive dyes resulted in good fastness properties and moderate unevenness in the dyed fabrics. The performance of direct dyes has been affected by competitive adsorption or poor compatibility with components in the PA extract, leading to irregular dye uptake. Reactive dyes, which require specific pH and temperature conditions to form covalent bonds with cellulose, likely underperformed due to interference from the PA matrix, which does not support such reactions. The variability in pH and competing functional groups may have inhibited proper dye–fibre interaction, leading to a patchy appearance and low shade reproducibility. These observations suggest that not all synthetic dyes are compatible with natural dye matrices, and interactions between dye types and fabric require careful control of conditions to achieve consistent results.

### 3.4. Fourier-transform infrared analysis

**Figure 9.**
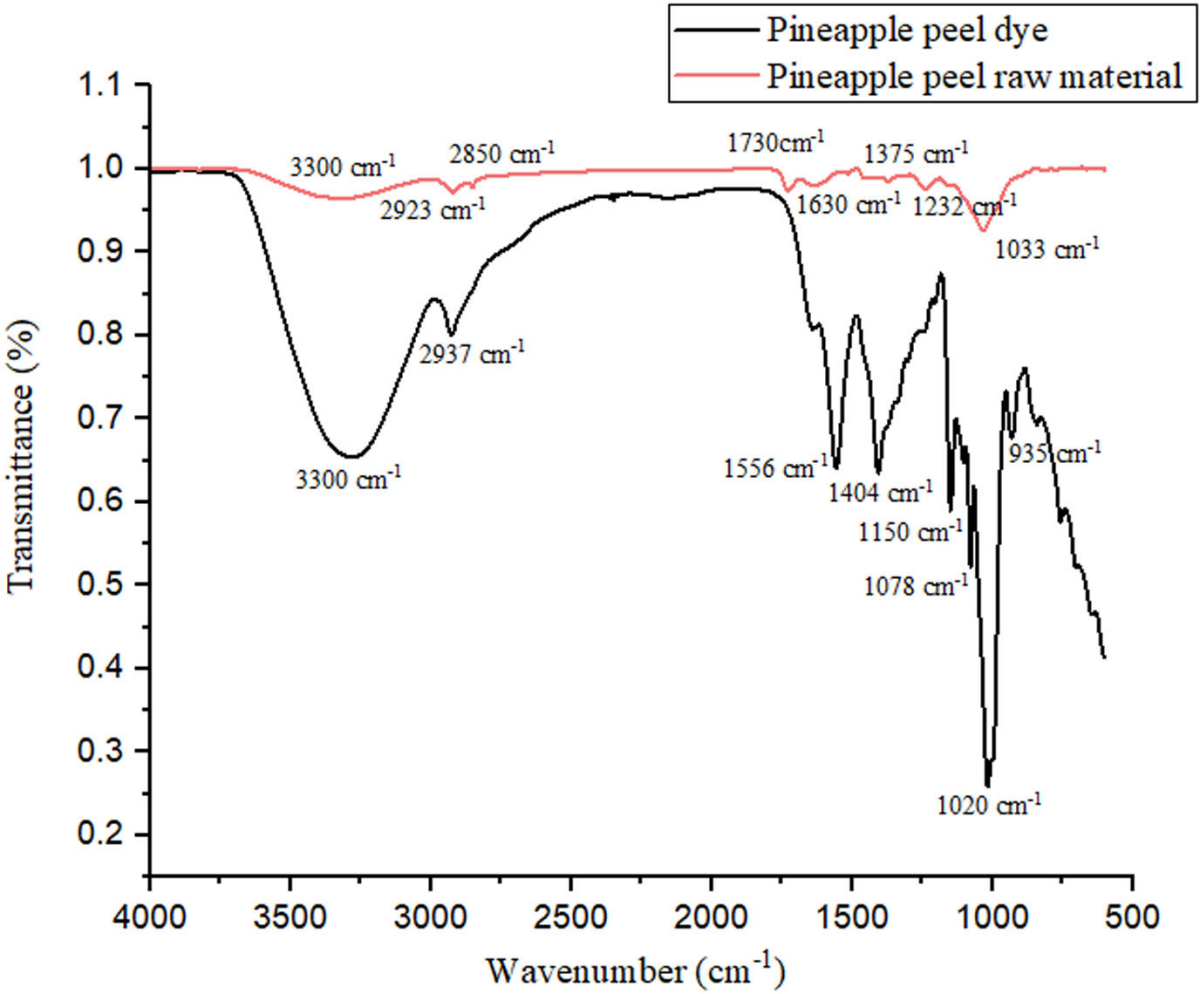
FTIR spectra of pineapple peel dye and dried pineapple peel raw material.

FTIR spectroscopy was conducted to identify the functional groups present in the dried pineapple peel and the PA dye, highlighting the molecular changes during the dye extraction process (figure 9). For the dried pineapple peels, a broad absorption band around 3300 cm^−1^ was observed, corresponding to O–H stretching, indicative of the hydroxyl groups present in polysaccharides and moisture. Peaks at 2923 cm^−1^ and 2850 cm^−1^ were attributed to C‒H stretching vibrations of aliphatic chains, while a prominent band at 1730 cm^−1^ was assigned to C=O stretching of carbonyl groups, likely from esterified compounds. Peaks were observed near 1600–1500 cm^−1^ for aromatic C=C stretching and 1000–1200 cm^−1^ for C–O stretching.

The FTIR spectrum of the PA dye powder revealed significant differences, reflecting structural modifications during the extraction process. The broad O–H stretching band around 3300 cm^−1^ exhibited increased intensity, suggesting a higher concentration of hydroxyl-containing compounds in the dye. The C=O stretching band at 1730 cm^−1^ showed a slight shift and reduction in intensity, indicating chemical transformations and possible conjugation of carbonyl groups. Enhanced intensities of peaks near 1600 and 1500 cm^−1^ confirmed the release of aromatic compounds, including phenolics and tannins, from the peel during extraction. The changes in the C‒O stretching region (1000–1200 cm^−1^) reflected the breakdown of polysaccharides and the presence of glycosides in the dye solution.

### 3.5. Thermogravimetric analysis of dye powder

**Figure 10.**
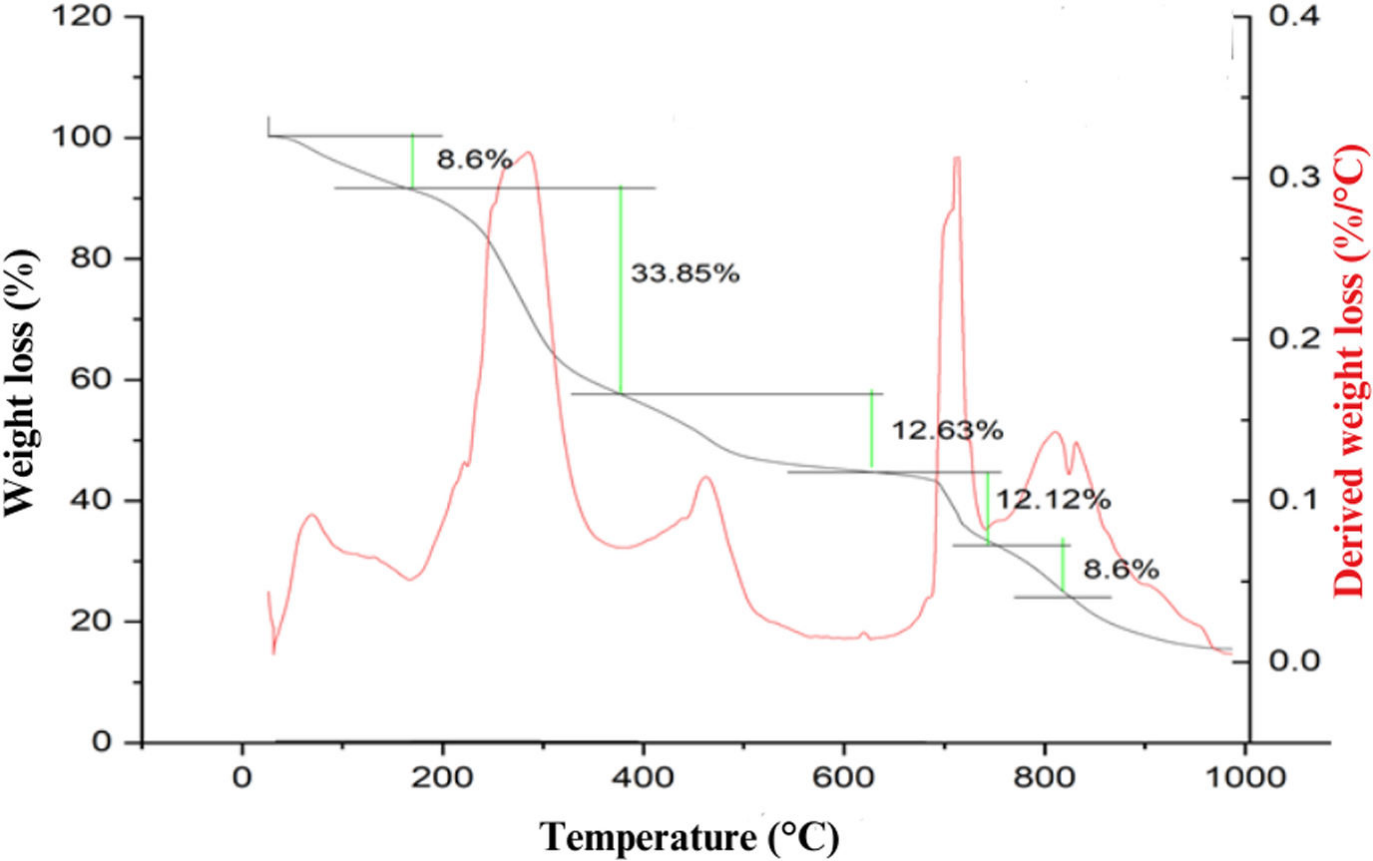
Thermogravimetric and differential thermogravimetric curves for pineapple peel dye.

The thermal stability of the pineapple peel dye was evaluated using thermogravimetric analysis (TGA) and differential thermogravimetric analysis (DTG), as shown in figure 10. These analyses provide insights into the thermal decomposition behaviour and the stability of the dye under high-temperature conditions. The TGA curve shows a multi-step weight loss pattern, with the following stages of thermal degradation identified. Initial weight loss (30–160°C): a small weight loss of approximately 8.6% was observed in this temperature range, attributed to the evaporation of moisture and the removal of physically adsorbed water molecules. This indicates that the dye powder is thermally stable up to 160°C, making it suitable for dyeing processes involving elevated temperatures without significant decomposition. In the second decomposition stage (160–400°C), a major weight loss of 33.85% occurred, which can be attributed to the degradation of organic components and represents the breakdown of thermally labile compounds present in the PA dye. Third decomposition stage (400–650°C): a further weight loss of 12.63% was observed in this range, corresponding to the thermal degradation of more stable organic compounds, such as carbonized residues or complex aromatic structures. In the final stage (650–900°C), a gradual weight loss of 12.12% was noted, likely due to the breakdown of residual carbonaceous material. Beyond 900°C, minimal weight change was observed, suggesting the completion of the thermal decomposition process. The DTG curve provides additional information on the rate of decomposition, where the peak decomposition temperatures corresponding to the major weight loss stages were identified, highlighting the most thermally labile regions.

### 3.6. Particle size analysis of dye powder

**Figure 11.**
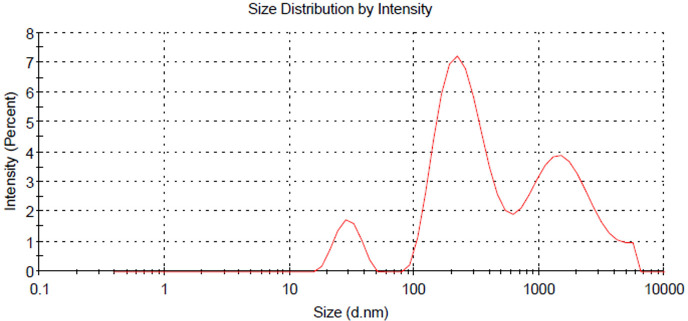
Particle size distribution of pineapple peel dye powder.

The particle size distribution of the PA dye powder was analysed, and the results are presented in figure 11. The analysis reveals two prominent average particle sizes, 266.2 and 1751 nm, indicative of a heterogeneous population of dye particles. This particle size is advantageous as it facilitates deep penetration into fabric fibres, enhancing the bonding of dye molecules more efficiently. Consequently, the dye exhibits vibrant and long-lasting colouration on both natural and synthetic fabrics. The small and uniform particle size distribution of the primary peak supports efficient solubility and dispersion of the dye in aqueous dyeing solutions. This property reduces processing time and ensures even colouration across the fabric surface, leading to improved dyeing efficiency, fastness properties and product consistency.

### 3.7. Mechanistic considerations in cotton dyeing

**Figure 12.**
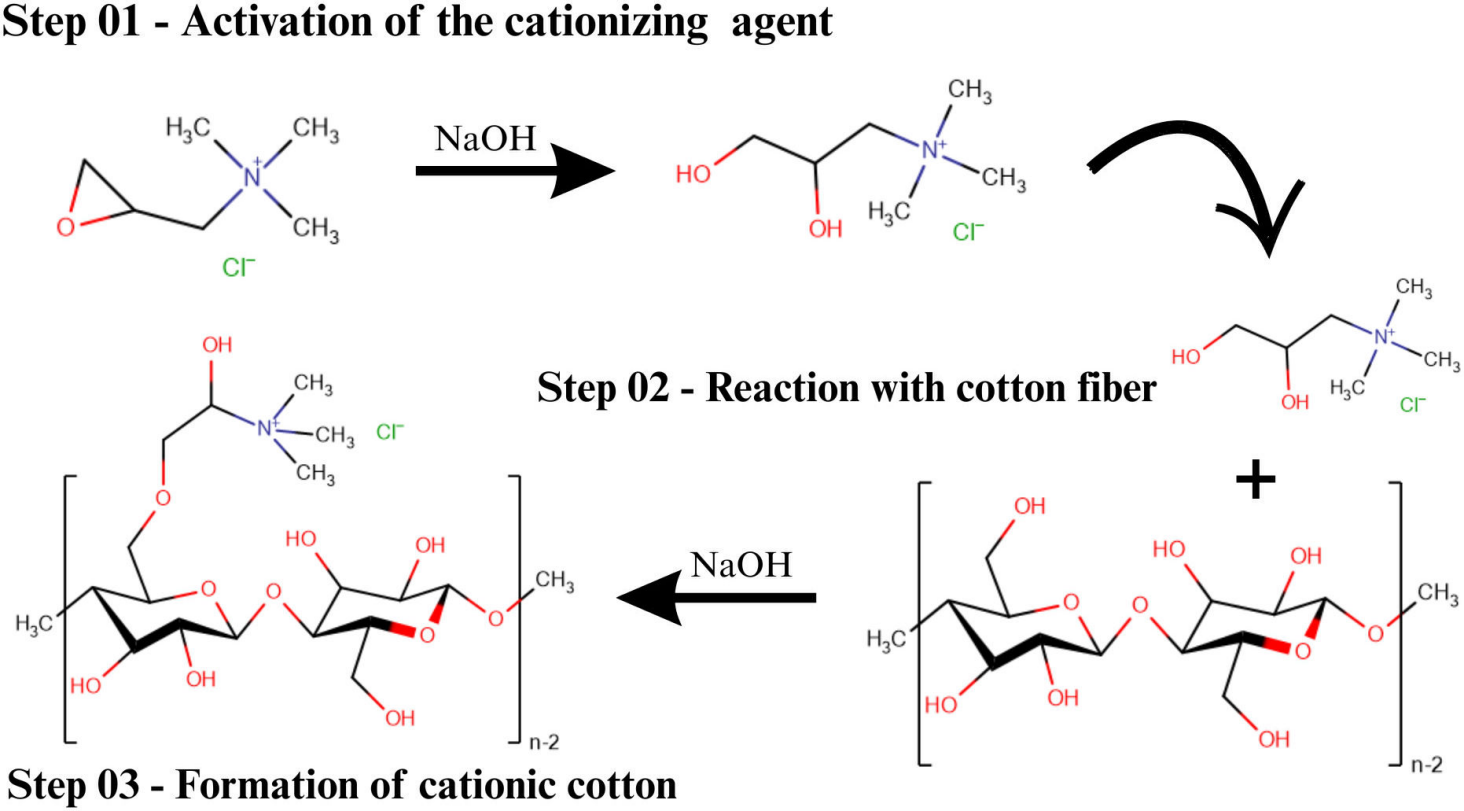
Schematic representation of the chemical modification of cotton fibres with a non-toxic cationizing agent [38].

Cotton fibres are primarily composed of cellulose, a linear polysaccharide with a high density of hydroxyl (–OH) groups, which impart hydrophilicity and a negative surface charge in aqueous solutions that reduces the fibre’s affinity for anionic dyes due to electrostatic repulsion. Natural dyes, such as polyphenolic compounds extracted from pineapple peel, typically contain phenolic –OH or carboxylic –COOH groups that dissociate under neutral to basic pH, further increasing dye anionicity. When cationizing agents are applied to cotton fibres, the mechanism of chemical modification significantly enhances the dyeing process, with reactive or direct dyes which are generally anionic in nature. Cationizing agents address this limitation by introducing positively charged groups, such as quaternary ammonium or protonated amines, onto the cellulose backbone (figure 12, step 01).

The process begins with the reaction of the cationizing agent with the hydroxyl groups in cellulose, resulting in covalent bonding through substitution or addition reactions (figure 12, step 02). For instance, quaternary ammonium compounds react through alkylation or substitution reactions, while epoxy-based agents form ether linkages with the cellulose. Polyamines provide multiple amine groups that protonate in acidic or neutral conditions, creating a permanent positive surface charge. This modification transforms the negatively charged cellulose into a cationic substrate, allowing strong electrostatic attraction between the fibre and anionic dye molecules.

Once the fibre is cationized, the positive charge on the fibre surface attracts negatively charged dye molecules, leading to enhanced dye adsorption and reduced dye loss during rinsing (figure 12, step 03). Unlike conventional dyeing processes, which often require high concentrations of salt to neutralize the fibre’s negative charge, cationized fibres can achieve higher dye uptake with minimal or no electrolyte addition, which not only reduces the environmental impact of the process but also improves the efficiency and cost-effectiveness of dyeing [34].

The cationization process also impacts the physical properties of the fibre. It increases the fibre’s swelling capacity in water, improving dye diffusion into the amorphous regions of cellulose. The strong electrostatic attraction between the dye and the modified fibre improves dye retention during washing. As such, the improved dyeing performance observed is primarily due to non-covalent interactions, including ionic attractions and hydrogen bonding.

**Figure 13.**
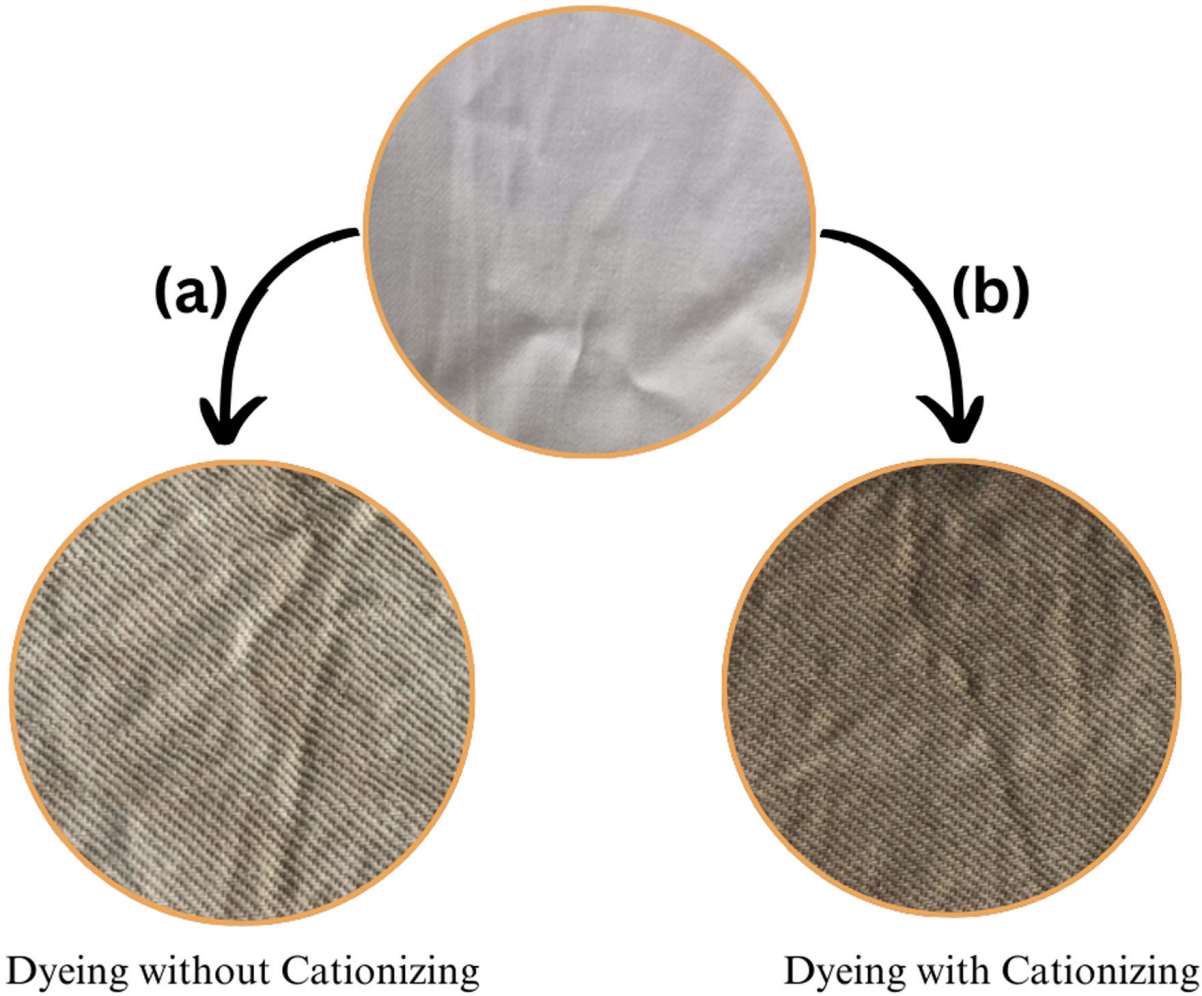
(a) Dyeing of cotton fabric with pineapple peel dye without cationizing. (b) Dyeing of cationized cotton fabric using Besol OED cationizing agent.

The cationized cotton fabric exhibits higher dye uptake and colour depth due to these enhanced electrostatic and physical interactions, as shown in figure 13. A comparative analysis was conducted on cotton fabrics dyed with PA dye under two conditions. In the first condition, direct PA dyeing without cationizing (figure 13a) resulted in pale, uneven colouration, which can be attributed to poor dye uptake caused by the inherent electrostatic repulsion between negatively charged cotton fibres and the anionic dye molecules. In contrast, dyeing with cationization using Besol OED (figure 13b) produced vibrant, uniform colouration with significantly enhanced colour depth. This result highlights the mechanistic role of cationization in modifying fibre surface charge, enabling ionic bonding with anionic dye molecules, and improving dye distribution and retention on cotton.

### 3.8. Dye exhaustion and fixation efficiency

The quantitative analysis of dyeing performance revealed significant insights into the interaction between PA dye and cotton cellulose. The dye exhaustion (%*E*) achieved 43.05%, indicating that approximately 43% of the dye initially present in the bath was adsorbed by the cotton fabric during the dyeing process. This falls within the moderate range typically observed for natural dyes on cellulosic substrates. This level of exhaustion indicates reasonable dye–fibre affinity, considering that natural dyes generally exhibit lower substantivity compared to synthetic counterparts due to their reliance on weaker non-covalent interactions such as hydrogen bonding and van der Waals forces with cellulose hydroxyl groups.

The total fixation efficiency (%*T*) was determined to be 27.76%, representing the proportion of the original dye that remained permanently bound to the fibres after the comprehensive wash-off procedure. This value, while moderate, is characteristic of natural dyeing systems where dye fixation depends primarily on physical adsorption and hydrogen bonding mechanisms rather than strong covalent bonds. The difference between exhaustion (43.05%) and total fixation (27.76%) indicates that approximately 15.29% of the initially absorbed dye was removed during the wash-off procedure, suggesting the presence of loosely bound or surface-adsorbed dye molecules.

The fixation ratio (%*F*) was calculated as 0.64, demonstrating that 64% of the exhausted dye successfully formed stable interactions with the cotton substrate, while 36% remained loosely associated and was subsequently removed during washing. This fixation ratio is considered acceptable for natural dyes, particularly when applied without mordants, as it demonstrates that a substantial majority of the absorbed colourant achieved sufficient binding strength to resist removal under standard washing conditions. The observed values are consistent with previous studies on natural dyes, where exhaustion rates typically range from 30% to 60% and fixation efficiencies from 20% to 40% for non-mordanted systems.

### 3.9. Fastness properties of the dyed fabrics

#### 3.9.1. Colour fastness to washing and laundering

**Figure 14.**
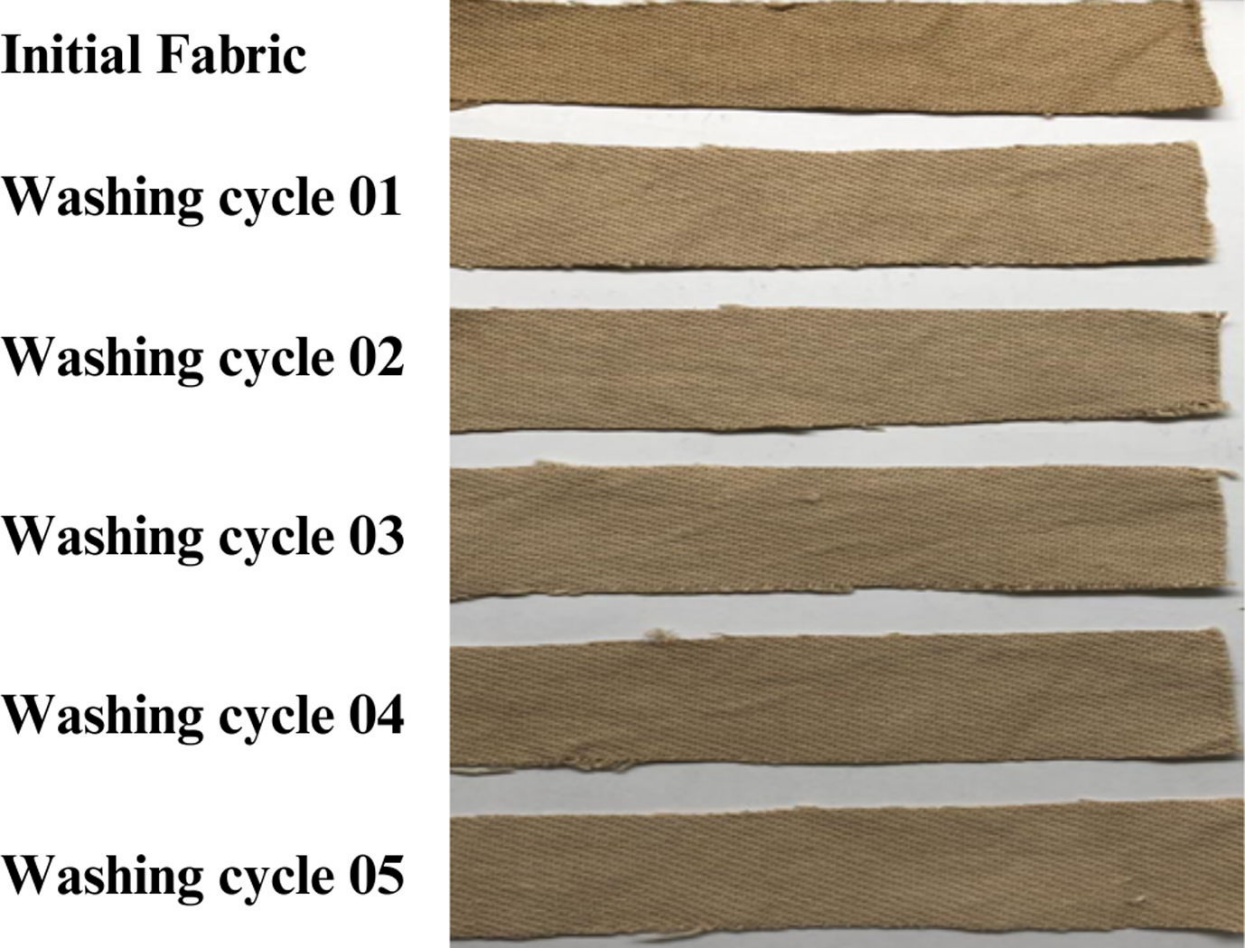
Colour fastness to wash of direct PA-dyed cotton fabric using ZnSO_4_ mordanting treatment, showing progressive colour change through successive washing cycles.

The fastness of washing and staining was evaluated using a grey scale ranging from 1 to 5, where 1 represents poor fastness and 5 represents excellent fastness (table 2). The colour fastness to washing of direct PA-dyed fabrics was evaluated over five washing cycles, as shown in figure 14. Variations in wash fastness were observed depending on the mordant used. Zinc sulfate post-mordanting showed excellent wash fastness (4–5), outperforming other mordants, likely due to its ability to form coordination complexes with hydroxyl or carboxylic groups in the dye molecules, reducing dye solubility and wash-off. Similarly, tannic acid post-mordanting exhibited good wash fastness (4), which can be attributed to the polyphenolic structure of tannic acid forming multiple hydrogen bonds with cellulose and metal ions, effectively anchoring the dye onto the fibre surface. Pre-mordanting with alum and ammonium ferrous sulfate resulted in poor ratings (1–3), emphasizing the importance of post-mordanting in enhancing dye retention during laundering. The improved durability can be attributed to reduced dye desorption caused by the formation of metal–dye or tannin–dye complexes, which exhibit lower aqueous solubility compared to unbound dye.

#### 3.9.2. Colour fastness to rubbing

Dry and wet rubbing fastness tests were conducted on fabrics mordanted with various agents, including alum, zinc sulfate and tannic acid (table 2). Zinc sulfate post-mordanting provided excellent results (4), indicating stable physical adherence of dye molecules on the fibre surface, possibly through coordination bonding. Conversely, pre-mordanting with alum and ammonium ferrous sulfate yielded moderate to poor ratings (2–3), indicating moderate resistance to mechanical stress in moist conditions. Tannic acid post-mordanting also performed well, with dry and wet rubbing ratings of 4 and 3–4, respectively, due to its ability to act as a natural mordant by forming hydrogen bonds and π–π interactions with the aromatic rings in the dye and cellulose.

#### 3.9.3. Colour fastness to light

**Figure 15.**
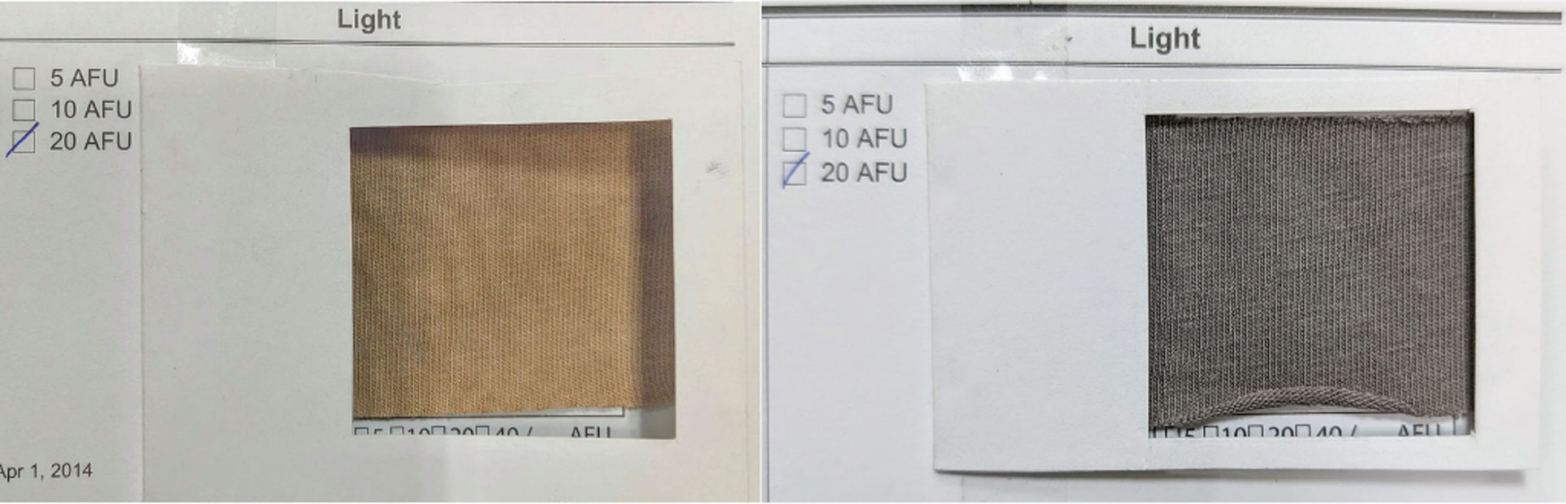
Sample specimen preparation for lightfastness testing using xenon arc lamp.

The lightfastness ratings show a clear distinction between the dye sources and mordanting methods and are summarized in table 2. PA dye alone exhibited the lowest rating (1), indicating significant fading under light exposure. The addition of KMnO_4_ resulted in a slight improvement in lightfastness rating from 2 to 3, likely due to the removal of loosely bound dye particles during the oxidative treatment process. However, simultaneous mordanting with tannic acid or post-mordanting with alum further enhanced ratings to 3 and 2, respectively, due to the formation of more stable dye–mordant complexes that protect against photodegradation by reducing the rate of photoinduced bond cleavage. Zinc sulfate post-mordanting achieved the highest lightfastness rating (4), suggesting the formation of metal–dye coordination bonds that stabilize the chromophore system against UV-induced fading. Figure 15 illustrates the sample preparations for the lightfastness test showcasing the standardized approach taken to ensure consistency and reliability of the results.

#### 3.9.4. Colour fastness to perspiration

Under acidic and alkaline conditions, post-mordanting with tannic acid and zinc sulfate consistently produced higher ratings (4), reflecting that these agents improve dye retention under ionic or pH-variable environments, by forming less soluble dye complexes (table 2). Alum and ammonium ferrous sulfate pre-mordanting resulted in lower ratings (2–3), suggesting weaker dye adherence due to insufficient complexation or poor penetration during pre-treatment. Overall data demonstrate that post-mordanting methods, particularly with zinc sulfate and tannic acid, significantly enhance fastness properties across various parameters.

#### 3.9.5. Absorption spectra analysis

**Figure 16.**
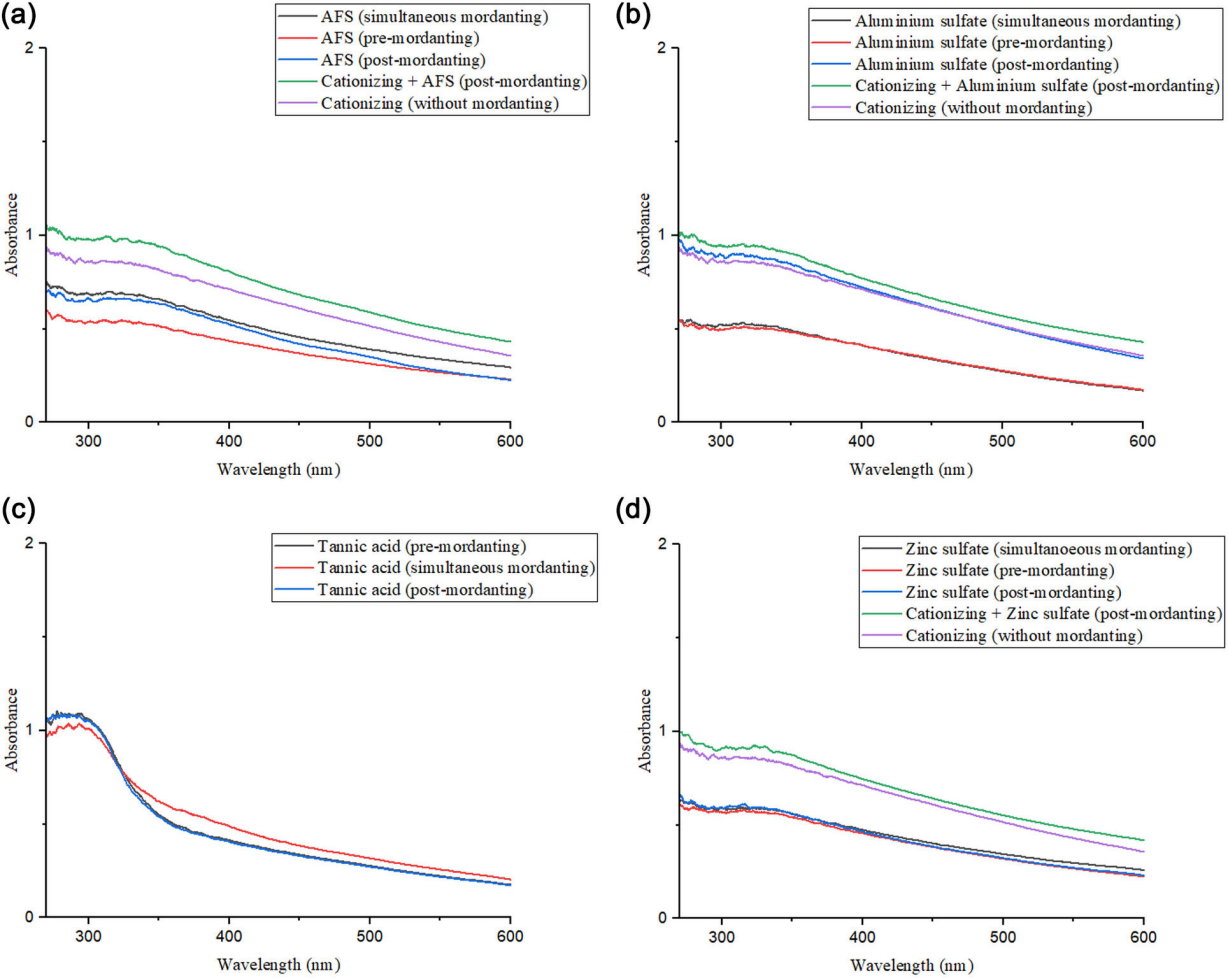
UV–vis diffuse absorption spectra of dyed cotton fabrics under various conditions, showing the effects of different mordants on UV absorption: (a) ammonium ferrous sulfate, (b) alum, (c) tannic acid and (d) zinc sulfate.

The absorption spectra of dyed fabrics were recorded over the UV–vis wavelength range and are presented in figure 16a–d, revealing that cationized dyeing when combined with post-mordanting significantly enhances UV absorption, regardless of the mordant used. This improvement can be attributed to the formation of metal–dye coordination complexes, which improve the fabric’s ability to absorb UV radiation. Cationization introduces positive charges onto the fibre surface, which attract anionic dye molecules more efficiently and influence molecular orientation and dye packing. However, the chemical structure of the cationizer plays a crucial role in determining UV stability [35]. Cationizers with aliphatic chains can interfere with the bonding between the dye and fabric due to their electron cloud distribution, leading to weaker interactions and higher dye degradation under UV exposure [39]. Cationizers containing aromatic rings are preferred to overcome this, as their rigid structure facilitates π–π stacking with dye molecules, reducing dye degradation and enhancing UV absorption. Among the tested methods, cationization—both with and without post-mordanting—consistently demonstrated the highest UV absorption, particularly when AFS and zinc sulfate were employed as mordants. This enhancement is primarily due to the improved electrostatic attraction between cationic fibres and anionic dye, in combination with stable metal–dye coordination that resists UV-induced breakdown. The results suggest that optimizing both the cationizing agent’s structure and mordanting conditions can effectively maximize UV absorption, improving the fabric’s protective properties against UV radiation.

#### 3.9.6. Colour measurements and statistical analysis of mordanting effects on pineapple peel dye performance

The descriptive statistical analysis revealed significant variations in colour strength and colorimetric parameters across different mordant types (table 7). Tannic acid demonstrated superior performance with the highest mean *K*/*S* value of 21.4 ± 10.7, which was substantially higher than that of all metallic mordants tested. This represents a 6.1-fold increase compared to zinc sulfate (*K*/*S* = 3.52 ± 5.00), 10.6-fold increase compared to alum (*K*/*S* = 2.01 ± 1.40) and 11.0-fold increase compared to ammonium ferrous sulfate (*K*/*S* = 1.94 ± 1.36). The exceptional performance of tannic acid can be attributed to its polyphenolic structure, which provides multiple binding sites for dye molecules through hydrogen bonding and coordination interactions, leading to enhanced dye uptake and colour intensity.

**Table 7.** Descriptive statistical analysis of colorimetric parameters and colour strength for different mordant types.

	mordant type	L*	a*	b*	*K*/*S*
N(?)	alum	4	4	4	4
	ammonium ferrous sulfate	4	4	4	4
	zinc sulfate	4	4	4	4
	tannic acid	3	3	3	3
mean	alum	−8.14	4.39	12.1	2.01
	ammonium ferrous sulfate	−9.57	3.64	11.4	1.94
	zinc sulfate	−9.30	3.29	9.99	3.52
	tannic acid	−7.52	3.87	10.4	21.4
standard deviation	alum	2.00	1.24	2.80	1.40
	ammonium ferrous sulfate	2.30	1.12	2.92	1.36
	zinc sulfate	2.09	0.926	2.23	5.00
	tannic acid	0.832	0.380	1.24	10.7
variance	alum	4.00	1.54	7.86	1.97
	ammonium ferrous sulfate	5.31	1.26	8.52	1.86
	zinc sulfate	4.35	0.857	4.98	25.0
	tannic acid	0.693	0.144	1.55	114
minimum	alum	−11.1	3.73	10.7	0.770
	ammonium ferrous sulfate	−12.8	2.74	8.64	0.880
	zinc sulfate	−12.4	2.53	8.11	0.840
	tannic acid	−8.47	3.53	9.08	13.8
maximum	alum	−7.00	6.25	16.4	3.41
	ammonium ferrous sulfate	−7.65	5.11	14.6	3.94
	zinc sulfate	−7.99	4.59	13.2	11.0
	tannic acid	−6.92	4.28	11.5	33.6

The colorimetric analysis in the CIELab colour space coordinates revealed distinct colour characteristics among different mordant treatments, with all L* values measured relative to undyed white cotton fabric as the control (table 8). Tannic acid exhibited the highest L* value (−7.52 ± 0.832), indicating the least darkening effect compared to metallic mordants, which showed greater darkening with L* values ranging from −8.14 to −9.57. The negative L* values across all treatments confirm successful dye uptake, with more negative values indicating greater colour depth relative to the white cotton control. The a* coordinate values were relatively consistent across all treatments (3.29–4.39), suggesting minimal variation in red–green chromaticity. However, b* values showed more variation, with alum treatments displaying the highest yellowness (12.1 ± 2.80), while zinc sulfate showed the lowest (9.99 ± 2.23). This variation in yellow chromaticity can be attributed to the different coordination chemistry of metal ions with PA dye chromophores, affecting the electronic transitions responsible for colour perception.

**Table 8.** Colorimetric parameters and colour strength values of cotton fabrics dyed with PA dye using different mordanting treatments.

treatment	mordant type	mordant method	L*	a*	b*	*K*/*S*
cationized + alum (post-mordanting)	alum	cationized + post-mordanting	−11.14	6.25	16.35	3.41
cationized + ammonium ferrous sulfate	ammonium ferrous sulfate	cationized + post-mordanting	−12.83	5.11	14.59	3.94
cationized + zinc sulfate (mordanting)	zinc sulfate	cationized + post-mordanting	−12.4	4.59	13.19	11.02
alum (pre-mordanting)	alum	pre-mordanting	−7	3.75	10.69	0.84
alum (simultaneous mordanting)	alum	simultaneous mordanting	−7.27	3.83	10.79	0.77
alum (post-mordanting)	alum	post-mordanting	−7.17	3.73	10.75	3.03
zinc sulfate (pre-mordanting)	zinc sulfate	pre-mordanting	−8.16	3.28	9.72	0.84
zinc sulfate (simultaneous mordanting)	zinc sulfate	simultaneous mordanting	−8.65	2.53	8.11	1.07
zinc sulfate (post-mordanting)	zinc sulfate	post-mordanting	−7.99	2.74	8.94	1.16
ammonium ferrous sulfate (pre-mordanting)	ammonium ferrous sulfate	pre-mordanting	−7.65	2.74	8.64	0.88
ammonium ferrous sulfate (simultaneous mordanting)	ammonium ferrous sulfate	simultaneous mordanting	−9.52	2.79	9.25	1.55
ammonium ferrous sulfate (post-mordanting)	ammonium ferrous sulfate	post-mordanting	−8.3	3.91	13.17	1.39
tannic acid (pre-mordanting)	tannic acid	pre-mordanting	-7.17	3.53	9.08	16.75
tannic acid (simultaneous mordanting)	tannic acid	simultaneous mordanting	-8.47	4.28	11.54	13.77
tannic acid (post-mordanting)	tannic acid	post-mordanting	-6.92	3.8	10.63	33.6

d*L*: lightness difference relative to undyed white cotton control; a*: red–green coordinate; b*: blue–yellow coordinate; *K*/*S*: colour strength calculated using Kubelka–Munk equation.

The descriptive statistical analysis revealed significant variations in colour strength and colorimetric parameters across different mordant types (table 7). Tannic acid demonstrated superior performance with the highest mean *K*/*S* value of 21.4 ± 10.7, which was substantially higher than that of all metallic mordants tested. This represents a 6.1-fold increase compared to zinc sulfate (*K*/*S* = 3.52 ± 5.00), 10.6-fold increase compared to alum (*K*/*S* = 2.01 ± 1.40), and 11.0-fold increase compared to ammonium ferrous sulfate (*K*/*S* = 1.94 ± 1.36). The exceptional performance of tannic acid can be attributed to its polyphenolic structure, which provides multiple binding sites for dye molecules through hydrogen bonding and coordination interactions, leading to enhanced dye uptake and colour intensity.

The colorimetric analysis in the CIELab colour space revealed distinct colour characteristics among different mordant treatments, with all L* values measured relative to undyed white cotton fabric as the control (table 8). Tannic acid exhibited the highest L* value (−7.52 ± 0.832), indicating the least darkening effect compared to metallic mordants, which showed greater darkening with L* values ranging from −8.14 to −9.57. The negative L* values across all treatments confirm successful dye uptake, with more negative values indicating greater colour depth relative to the white cotton control. The a* coordinate values were relatively consistent across all treatments (3.29–4.39), suggesting minimal variation in red–green chromaticity. However, b* values showed more variation, with alum treatments displaying the highest yellowness (12.1 ± 2.80), while zinc sulfate showed the lowest (9.99 ± 2.23). This variation in yellow chromaticity can be attributed to the different coordination chemistry of metal ions with PA dye chromophores, affecting the electronic transitions responsible for colour perception.

The range analysis further supports these findings, with tannic acid showing the widest *K*/*S* range (13.8–33.6), confirming that the mordanting method significantly influences performance when using this bio-mordant. Among metallic mordants, zinc sulfate demonstrated the broadest *K*/*S* range (0.84–11.0), indicating that this mordant’s effectiveness is highly dependent on application conditions. The minimum *K*/*S* values across all treatments (0.77–0.88) were remarkably similar, suggesting a baseline dye uptake independent of mordant type, while the maximum values clearly distinguished the superior performance of tannic acid and zinc sulfate treatments.

These statistical findings align with recent research emphasizing the potential of bio-mordants as sustainable alternatives to heavy metals in textile dyeing. The resulting colour strength (*K*/*S*) of the bio-mordant pre-treated sample was two times higher (*K*/*S* = 8.6) than that of the metal-mordanted sample (*K*/*S* = 4.0), supporting the observation that tannic acid significantly outperformed metallic mordants. The enhanced performance of tannic acid can be explained by its ability to form stable complexes with PA dye molecules through multiple interaction mechanisms, including hydrogen bonding, π–π stacking, and coordination bonding, resulting in superior colour strength and potentially improved fastness properties. These results demonstrate that tannic acid represents a viable and environmentally sustainable alternative to traditional metallic mordants, offering superior colour strength while maintaining acceptable colour coordinate consistency. The high coefficient of variation observed in tannic acid *K*/*S* values suggests that optimization of mordanting conditions could further enhance performance uniformity, making it an attractive option for industrial-scale natural dyeing applications where both environmental sustainability and colour quality are priorities.

## 4. Conclusion

This study examined the feasibility of using pineapple peel, an agro-industrial by-product, as a sustainable and biodegradable natural dye source for cotton textile applications through systematic optimization and quantitative performance evaluation. Statistical modelling using four-factor linear regression identified alkaline extraction as the critical parameter (*p* = 0.046), with the optimized extraction conditions (MLR of 1 : 10, 12% NaOH concentration, 80°C, and a 2 h duration) yielding a pigment extraction efficiency of 35–40%. This outperformed the yields of less than 1% achieved with other solvents such as hexane, ethanol, and methanol. HPLC analysis provided mechanistic insights, revealing that alkaline conditions enhanced polyphenolic derivative compound extraction, with increased compound diversity attributed to cell wall disruption and improved phenolic solubility through deprotonation mechanisms.

Quantitative dyeing performance metrics established the dye’s commercial potential, with measured exhaustion (43.05 %), total fixation efficiency (27.76%) and fixation ratio (0.64), demonstrating acceptable dye–fibre interaction for natural dyeing systems. Colorimetric evaluation revealed significant mordant-dependent variations, with bio-mordant tannic acid achieving exceptional colour strength (*K*/*S* = 21.4 ± 10.7), representing 6.1- to 11.0-fold improvements over metallic mordants. CIELab coordinate analysis (L* = −6.92 to –12.83, a* = 2.53 to 6.25, b* = 8.11 to 16.35) confirmed successful chromophore fixation across all treatment conditions, with tannic acid providing optimal colorimetric balance.

Among the mordants tested, zinc sulfate post-mordanting produced superior wash fastness (4–5) compared to tannic acid (4) and alum (3). Lightfastness ratings improved from 1 (without mordant) to 4 with zinc sulfate, showcasing its ability to form stable dye–metal complexes that resist photodegradation. Cationized fabrics displayed an improvement in dye uptake compared to non-cationized dyeing, indicating the efficacy of cationization in mitigating electrostatic repulsion between the negatively charged cotton fabric and anionic dye molecules. UV absorption analysis further demonstrated that cationized fabrics, especially when post-mordanted with zinc sulfate or AFS, achieved the highest UV absorption, likely due to synergistic effects between electrostatic attraction and coordination complex formation.

The thermal stability characterization (stable to 160°C) and optimized particle size distribution (266.2 nm) support industrial processing compatibility, while the successful integration with existing textile finishing processes demonstrates scalability potential. Environmental benefits include waste valorization of agricultural by-products, elimination of synthetic colourant dependencies, and reduced processing chemical requirements through cationization-enabled salt-free dyeing protocols.

This research establishes a robust scientific foundation for pineapple peel-derived natural dyes in sustainable textile manufacturing, with quantified performance metrics supporting commercial feasibility. The integration of statistical optimization, advanced characterization techniques and comprehensive performance evaluation provides a replicable framework for natural dye development, contributing to circular economy initiatives and environmentally conscious textile production practices. Future research directions should focus on scaling optimization, life cycle assessment and exploration of additional functional properties to further enhance the commercial attractiveness of this sustainable dyeing technology.

## Data Availability

The datasets supporting this article have been uploaded as part of the electronic supplementary material [40].
